# Long noncoding RNA TRPM2-AS acts as a microRNA sponge of miR-612 to promote gastric cancer progression and radioresistance

**DOI:** 10.1038/s41389-020-0215-2

**Published:** 2020-03-02

**Authors:** Jian Xiao, Linling Lin, Dakui Luo, Liang Shi, Wangwang Chen, Hao Fan, Zengliang Li, Xiang Ma, Peidong Ni, Li Yang, Zekuan Xu

**Affiliations:** 1 Department of General Surgery, The First Affiliated Hospital of Nanjing Medical University, Nanjing, Jiangsu Province China; 2 Department of Colorectal Surgery, Fudan University Shanghai Cancer Center, 200032 Shanghai, China; 3 Department of General Surgery, Liyang People’s Hospital, Liyang Branch Hospital of Jiangsu Province Hospital, Liyang, Jiangsu Province China

**Keywords:** Cell migration, Gastric cancer, Cell growth

## Abstract

Long noncoding RNAs (lncRNAs) are emerging as important regulators of tumorigenesis and are frequently dysregulated in cancers. Here, we identify a critical lncRNA TRPM2-AS which is aberrantly expressed in gastric cancer (GC) tissues by screening The Cancer Genome Atlas Program(TCGA) database of GC cohort, and its upregulation is clinically associated with advanced pathologic stages and poor prognosis in GC patients. Silencing TRPM2-AS inhibits the proliferation, metastasis and radioresistance of GC cell whereas ectopic expression of TRPM2-AS significantly improves the progression of GC cell in multiple experiments. Mechanistically, TRPM2-AS serves as a microRNA sponge or a competitive endogenous RNA (ceRNA) for tumor suppressive microRNA miR-612 and consequently modulates the derepression of IGF2BP1 and FOXM1. Moreover, induced upregulation of IGF2BP1 subsequently increases the expression of c-Myc and promotes GC cell progression. Meanwhile, TRPM2-AS promotes the radioreistance of GC cell through enhancing the expression of FOXM1 as well. Thus, our findings support a new regulatory axis between TRPM2-AS, miR-612, IGF2BP1, or FOXM1 which serve as crucial effectors in GC tumorigenesis and malignant development, suggesting a promising therapeutic and diagnostic direction for GC.

## Introduction

Gastric cancer is one of the most common malignant cancer in the world and ranks the third most common causes of cancer mortality worldwide^1^. Due to its non-specific symptoms and highly rate of metastasis, GC patients are always diagnosed at advanced stage with poor prognosis, especially in China^2,3^. GC development is supposed as a stepwise process involving genetic and epigenetic alterations, such as TP53, MYC, KRAS, and PTEN^4^. However, the underneath molecular mechanism of GC carcinogenesis is still unclear and the practical diagnostic markers are lacking as well.

Recent years, accumulating data from whole genome and transcriptome studies have elucidated that the majority of human genome encodes massive numbers of non-coding RNAs^5,6^. Therefore, lncRNAs and microRNAs (miRNAs) which constitute the main part of non-coding RNAs have received widespread concern in cancer development^7–9^. LncRNAs are a class of non-coding RNAs that transcript longer than 200 nucleotides and have been implicated in regulating cancer cell proliferation, metastatic cascades, and chemoradiotherapy resistance via participating in post-transcription regulatory processes such as sponging miRNAs^10–12^. The activity of sponging microRNAs depends on microRNA response elements (MREs) presented in lncRNAs and downstream targets, in which lncRNAs compete with downstream targets to bind to microRNAs by MREs. Therefore it control one another expression through a decrease in microRNA detection and thus an impairment of microRNA activity^13^.

In case of gastric cancer, lncRNA expression profile is frequently dysregulated and several lncRNAs were involved in GC tumorigenesis such as GAPLING^14^, HOTAIR^15^, UCA1^16^. In this study, we intend to elucidate the oncogenic role of lncRNA TRPM2-AS in GC which acts as a microRNA sponge. Loss and gain of function assays showed that TRPM2-AS promotes GC cell proliferation, metastasis and radioresistance via functioning as ceRNA for tumor suppressor, miR-612. The insulin-like growth factor-2 mRNA-binding protein 1 (IGF2BP1), defined as RNA-binding, oncofetal protein, belongs to a conserved family which includes IGF2BP2 and IGF2BP3^17^. IGF2BP1 can regulate the expression of several oncogenic factors including c-Myc, IGF2, ACTB by affecting their stability, translatability, or localization^18^. We initially found that TRPM2-AS could sponge miR-612 and prevent its association with targeting IGF2BP1 mRNA, resulting in impaired c-Myc RNA degradation. Meanwhile, we also revealed TRPM2-AS could enhance the expression of the Forkhead Box M1(FOXM1) to promote the radioresistance of GC as ceRNA for miR-612. Thus, our results provide novel insights into the molecular mechanisms and potential therapeutic and diagnostic targets for GC.

## Results

### Upregulation of TRPM2-AS is associated with poor prognosis in GC

**Fig. 1:**
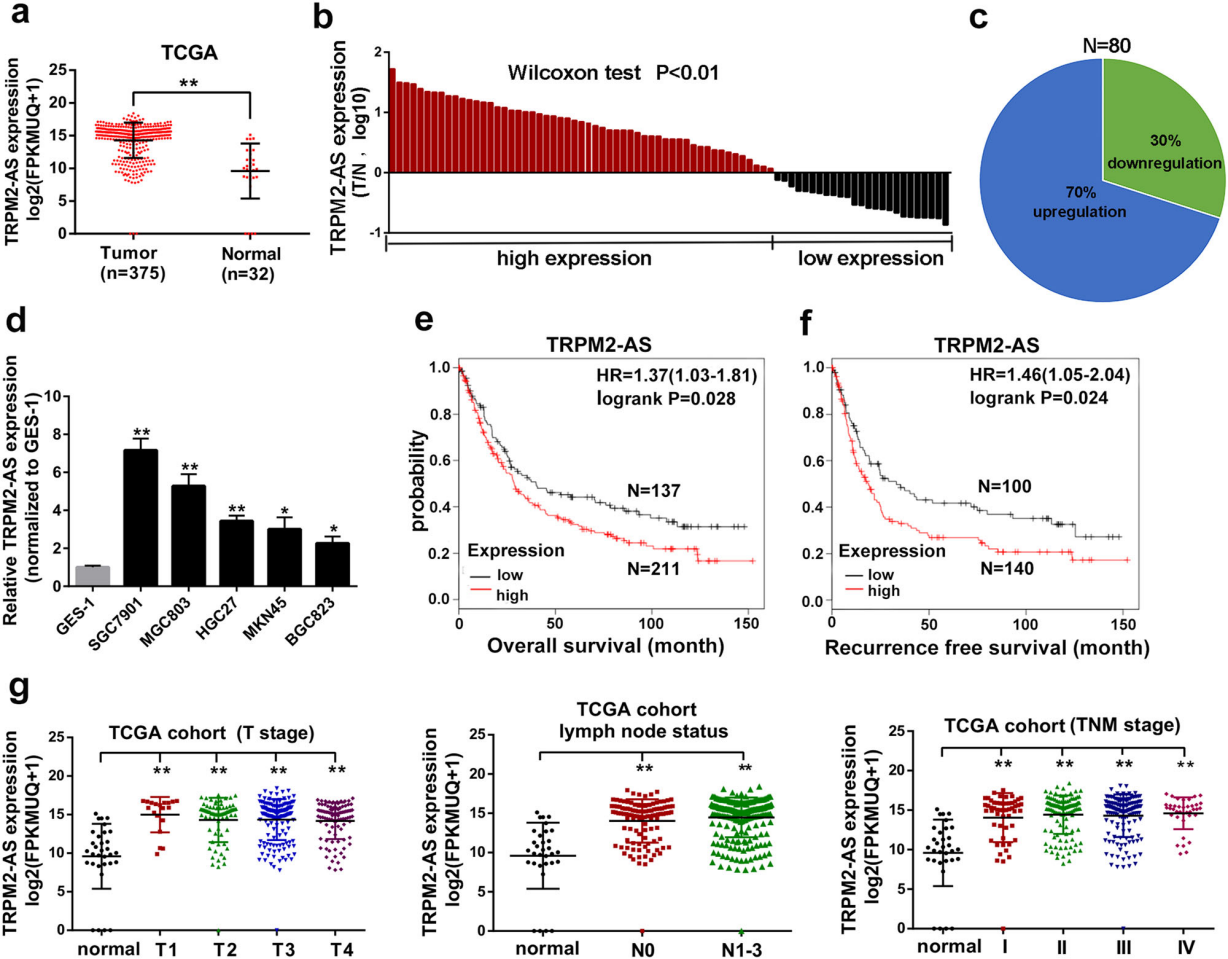
LncRNA TRPM2-AS is upregulated in GC and is associated with poor prognosis. **a** Relative expression of TRPM2-AS in GC tissues obtained from TCGA database. **b** The fold changes (log_10_) of TRPM2-AS in 80 paired GC tissues compared with adjacent normal tissues by qRT-PCR and were arranged from high to low. **c** The expression of TRPM2-AS was divided into two groups in 80 paired GC tissue samples. **d** qRT-PCR analysis of the expression of TRPM2-AS in GC cell lines and GES-1. **e** and **f** Kaplan–Meier OS and RFS curves according to the expression level of TRPM2-AS. **g** Expression patterns of TRPM2-AS based on T stage (left panel), N stage (middle panel) and TNM stage (right panel) from TCGA database. Error bars, mean ± SD. *P < 0.05; **P < 0.01.

To identify potential lncRNAs which participate in GC pathogenesis, we first explored the TCGA database and found lncRNA TRPM2-AS was highly expressed in GC tissue samples compared with normal tissues (Fig. 1a). Meanwhile, an analogous mode was also detected via measuring the expression of 80 paired gastric cancer tissues and adjacent normal tissues using reverse transcription and quantitative real-time PCR (qRT-PCR) (Fig. 1b). Among these paired samples, 70% (*n* = 56) of patients exhibited higher expression of TRPM2-AS in cancer tissues than in matched noncancerous tissues (Fig. 1c). Then we also detected higher expression of TRPM2-AS in GC cells compared with an immortalized gastric mucous epithelium cell line GES-1 (Fig. 1d). Collectively, all these data indicated that TRPM2-AS is upregulated in GC. In addition, in order to calculate the coding potential of TRPM2-AS, Coding-Potential Assessment Tool (CPAT)^19^ and Coding Potential Calculator (CPC)^20^ were applied. The results showed the coding probability of TRPM2-AS was very low (Fig. S1a, b). Meanwhile, ORF Finder analysis also failed to generate a protein of more than 165 amino acids and the lack of a valid Kozak sequence in TRPM2-AS further confirmed its non-coding feature (Fig. S1c)^11^.

GC patients were arranged by the expression level of TRPM2-AS, and the clinicopathologic characteristics were further analyzed. As shown in Supplementary Table 1, the expression level of TRPM2-AS was higher in T2–T4 stage than in Tis-T1 (*p* < 0.001). Meanwhile, patients with lymph node metastasis (*P* = 0.001) and higher TNM stages (*P* = 0.001) also presented with higher expression of TRPM2-AS. Moreover, analysis of TCGA cohort showed the upregulation of TRPM2-AS was associated with advanced overall stage and T/N stages as well (Fig. 1g). Notably, when we examined the prognostic values of TRPM2-AS in GC patients using online tool Kaplan Meier-plotter, it showed high expression level of TRPM2-AS accompanied with shorter overall survival rate (OS) and recurrence free survival (RFS) time in GC patients (Fig. 1e, f).

### Knockdown of TRPM2-AS inhibits GC cell growth

**Fig. 2:**
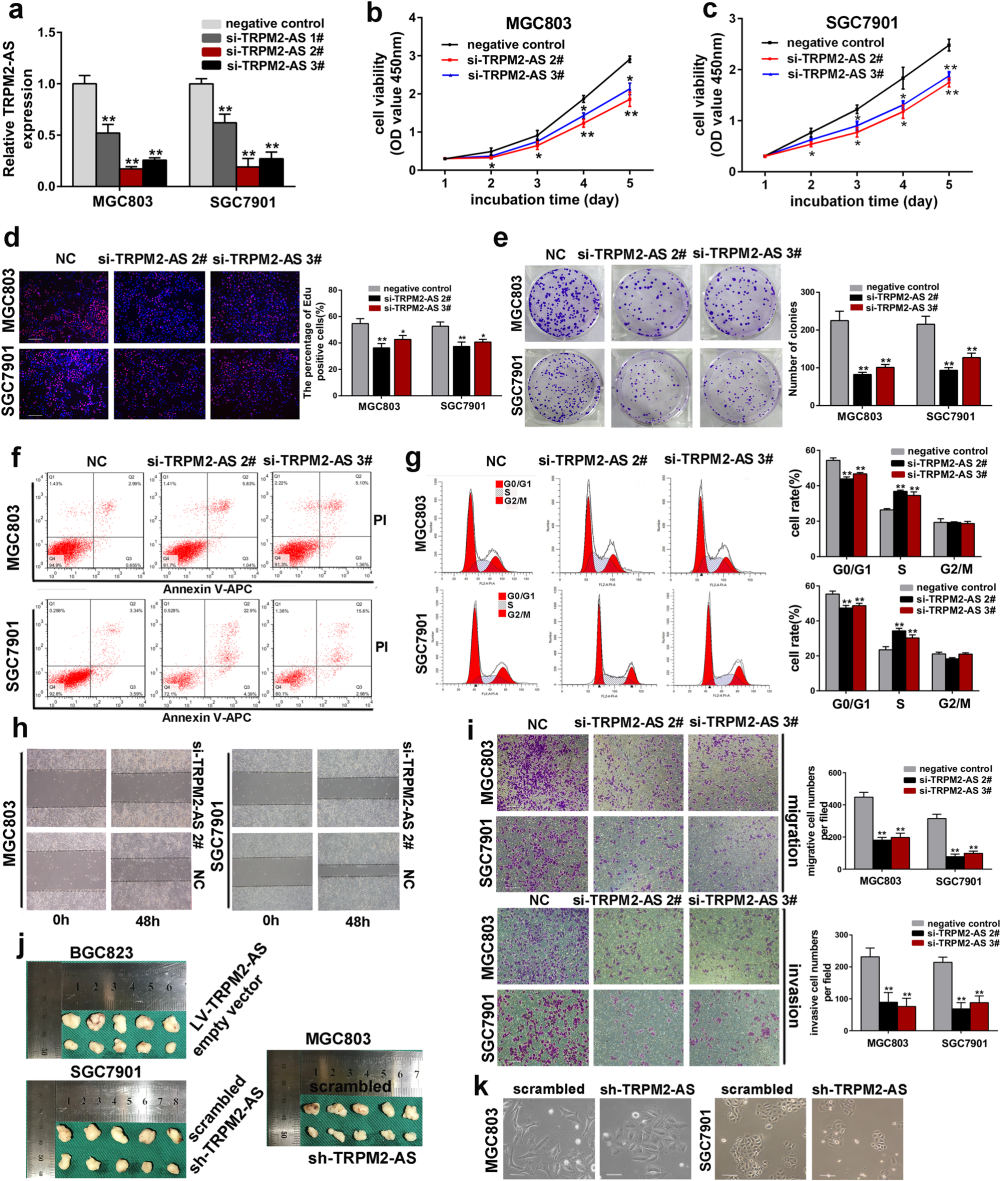
Effects of inhibiting the expression of TRPM2-AS on GC cell proliferation, migration and invasion. **a**. The expression of TRPM2-AS was downregulated in SGC7901 and MGC803 cells using siRNAs. **b**–**e** The proliferation of SGC7901 and MGC803 cells transfected with siRNA against TRPM2-AS (siRNA2#, siRNA3#) were measured using CCK8 assays (**b, c**), EdU incorporation assays, 100×, Scale bars = 100 μm (**d**), and colony formation assays (**e**). **f**, **g** Flow cytometric analysis of SGC7901 and MGC803 cells transfected with siRNAs or control about apoptotic rates (**f**) and cell cycle arrest (**g**). **h** Wound scratch assays for TRPM2-AS in GC cell migration. **i**. Transwell assays for TRPM2-AS in GC cell migration and invasion. 100×, Scale bars = 100 μm. **j** Tumor size after the tumors were harvested. **k** Typical images showing the morphological changes of MGC803 and SGC7901 cells treated with TRPM2-AS shRNA. Scale bars = 50 μm. Error bars, mean ± SD. *P < 0.05; **P < 0.01.

To investigate the effect of TRPM2-AS on GC tumorigenesis, SGC7901 and MGC803 cell lines which exhibit high expression of TRPM2-AS were transfected with siRNAs against TRPM2-AS (siRNA1#-3#; Fig. 2a). The growth curves detected by CCK8 assays showed knockdown of TRPM2-AS significantly inhibited the growth of GC cell (Fig. 2b, c). In accordance with the results of CCK8 assays, EdU incorporation assays presented that EdU positive cell numbers were drastically reduced following downregulation of TRPM2-AS (Fig. 2d). Moreover, colony formation assays displayed the same effects of silencing TRPM2-AS as presented in Fig. 2e. To further illustrate the oncogenic role of TRPM2-AS in vivo, we subcutaneously injected GC cells which were transduced with lentivirus vector contained TRPM2-AS-targeting shRNA sequence or scrambled sequence into the flank of 4 weeks female nude mice (Fig. 2j; Fig. S2a). It was obvious that the tumor formation rates in TRPM2-AS silencing groups were highly slowed compared with the control groups and the tumor weight in TRPM2-AS knockdown groups were also significantly reduced (Fig. S3h, i). There is no doubt that these data verified the essential role of TRPM2-AS in regulating GC cell proliferation.

As increased apoptosis and cell-cycle arrest are two factors that could contribute to the growth inhibition of cancer cell. Therefore, we assessed the effect of silencing TRPM2-AS on these properties by performing flow cytometric assays in GC cells. We found that knockdown of TRPM2-AS progressively increased the proportion of cells in the S phase to the detriment of the G0/1 phase, which confirmed the induction of cell cycle arrest at S phase after silencing TRPM2-AS (Fig. 2g). Furthermore, Annexin V-APC/PI staining showed the percentage of apoptotic cells were significantly increased by depleting TRPM2-AS (Fig. 2f; Fig. S2b, c). In line with the results generated above, the expression level of S-phase checkpoint proteins such as CyclinA2, CDK2 and anti-apoptosis proteins like Bcl2 and Bcl-xl were significantly reduced while pro-apoptosis protein like Bax was strongly enhanced after knockdown of TRPM2-AS (Fig. S2d). Taken together, these data uncovered that downregulation of TRPM2-AS impaired the growth of GC cell by inducing S-phase arrest and apoptosis.

Meanwhile, we overexpressed the expression of TRPM2-AS in BGC823 cells using lentivirus vector containing the 875 bp cDNA sequences of TRPM2-AS (Fig. S3a). Notably, multiple in vitro and in vivo experiments showed ectopic expression of TRPM2-AS could promote GC cell proliferation, migration and invasion (Fig. S3b–g, j).

### Downregulation of TRPM2-AS attenuates cell migration and invasion

Metastasis is a noteworthy hallmark of GC, so we delineate further about the functional significance of TRPM2-AS on GC cell migration and invasion. By carrying transwell assays with or without coating by matrigel, we examined the influence of silencing TRPM2-AS on the metastatic activity of GC cell. Significantly, the number of cells which getting through the chamber in knockdown group were much less than the control (Fig. 2i), and wound healing assay also showed depleting TRPM2-AS impaired GC cell migration (Fig. 2h). Furthermore, loss of TRPM2-AS increased the protein level of epithelial marker like E-cadherin whereas decreased the expression of mesenchymal markers such as N-cadherin and vimentin, indicating that TRPM2-AS may involve in the epithelial–mesenchymal transition of GC (Fig. S2d). Moreover, typical morphological changes of SGC7901 and MGC803 cells were noted after transduced with TRPM2-AS-targeting shRNA (Fig. 2k). Compared with wild type cells, cells treated with TRPM2-AS shRNA condensed and assumed a cobblestone-like morphology. Furthermore, we investigated the effect of TRPM2-AS on GC cell dissemination and metastatic colonization in vivo by inoculating GC cells directly into the tail vein of nude mice. It showed the lung metastatic burden was decreased in TRPM2-AS-silencing group compared with the control (Fig. S3k). Consequently, our results suggested that TRPM2-AS is essentially involved in the metastasis of GC.

### TRPM2-AS functions as sponge for miR-612 in GC cells

**Fig. 3:**
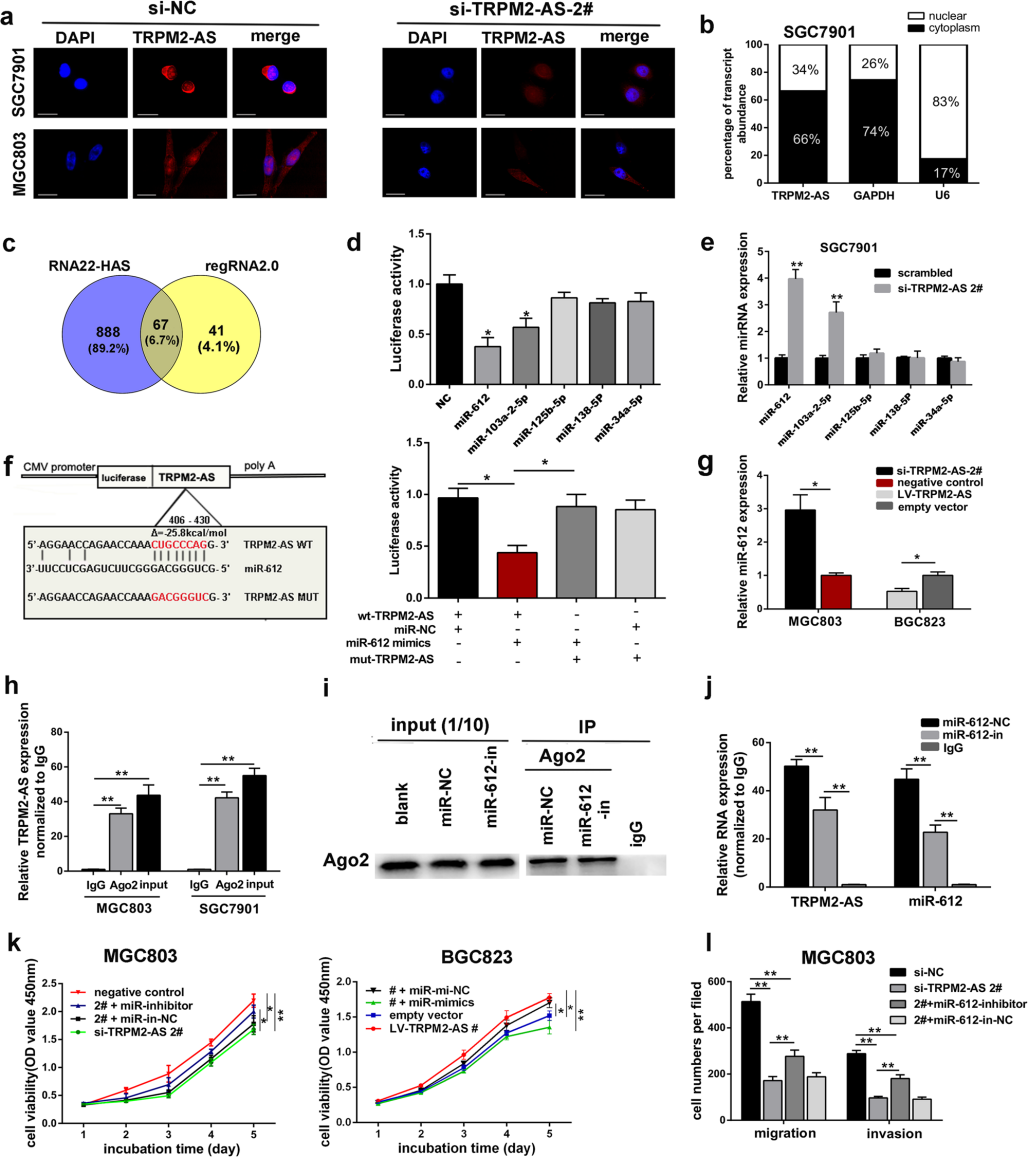
LncRNA TRPM2-AS serves as a sponge for miR-612. **a** RNA FISH assay revealed TRPM2-AS mainly located in the cytoplasm of GC cells. Nuclei was stained by DAPI as blue, TRPM2-AS sequence was labeled by Cy3 as red. Scale bars represent 25 μm. **b** Subcellular fractionation assay confirmed the location of TRPM2-AS in SGC7901 cells. **c** Venn diagram showed the downstream targets of TRPM2-AS by RNA22-HAS and RegRNA2.0. **d** The constructed luciferase reporter plasmid was cotransfected with miRNA mimics into HEK 293 T cells. Data are presented as the relative ratio of *renilla* luciferase activity to firefly luciferase activity. **e** The expression of five predicated miRNAs by qRT-PCR. **f** Luciferase report vectors contained mutated-type or wild-type sequence of TRPM2-AS were cotransfected with miR-612 mimics or control into HEK 293 T cells as indicated. **g** Relative expression of miR-612 by qRT-PCR. **h** Anti-Ago2 RIP was performed in GC cells and relative RNA level of TRPM2-AS in the immunoprecipitates was detected by qRT-PCR. **i** Ago2 protein was immunoprecipitated from SGC7901 cell extracts by anti-Ago2 RIP. **j** Anti-Ago2 RIP was performed in SGC7901 cells treated with miR-612 inhibitor and relative level of TRPM2-AS and miR-612 were detected. **k**–**i** TRPM2-AS requires miR-612 to promote GC cell proliferation by conducting CCK8 assays (**k**) and cell metastasis by transwell assays (**l**). Error bars, mean ± SD. *P < 0.05; **P < 0.01.

Recently, plenty of lncRNAs have been reported to exert their functions as ceRNA to sponge microRNAs. First, in situ hybridization assay and subcellular fractionation assay confirmed that TRPM2-AS mainly located in the cytoplasm of GC cell, suggesting TRPM2-AS might regulate the expression of targets at the post transcriptional level. (Fig. 3a, b). By using online tool RNA22-HAS and regRNA2.0, we observed that the sequence of TRPM2-AS contains potential miR-612, miR-103a-2-5p, miR-125b-5p, miR-138-5P, and miR-34a-5p binding sites with high scores (Fig. 3c). To further prove these predictions, we then conducted dual luciferase reporter assays. The results indicated that only overexpressing miR-612 and miR-103a-2-5p could reduce TRPM2-AS-driven luciferase activity, whereas the other 3 miRNAs failed to yield similar effect (Fig. 3d). Furthermore, qRT–PCR analysis indicated that the expression level of miR-612 was negatively regulated by TRPM2-AS (Fig. 3e, g). Since miR-612 exhibited the most evident change after silencing TRPM2-AS (Fig. 3e), so we exclusively investigated the relationship between TRPM2-AS and miR-612. Then, we engineered luciferase reporters containing mutated TRPM2-AS sequence which altered the potential binding sites for miR-612 and then transduced it into 293T cells with miR-612 mimics or control. As expected, the observed inhibition of luciferase activity by miR-612 mimics was abolished by the mutated TRPM2-AS sequence (Fig. 3f). In addition, RNA-binding protein immunoprecipitation(RIP) assay of gastric cancer cell extracts revealed that TRPM2-AS binds directly to Ago2 which is the key component of the RNA-induced silencing complex mediated by miRNA (Fig. 3h). Besides that, Ago2 protein was also successfully immunoprecipitated from cell extracts using Ago2 antibody (Fig. 3i). Furthermore, RIP assay demonstrated that compared with control group, the RNA level of TRPM2-AS and miR-612 were drastically decreased in Ago2 complexes purified from cells treated with miR-612 inhibitor, which further suggested TRPM2-AS may participate in miR-612-RISC complex (Fig. 3j). Moreover, the exact copy numbers of TRPM2-AS and miR-612 were quantified per cell by qRT-PCR to evaluate whether the abundance of TRPM2-AS were comparable to miR-612 for it to serve as a ceRNA. The result indicated that in SGC7901 cells, the expression level of TRPM2-AS was 899 copies per cell, whereas this of miR-612 was 1655 copies per cell, respectively. And through analyzing of 20 gastric cancer tissue samples, we found a significant inverse correlation between TRPM2-AS and miR-612 (*R* = −0.5253, *P* = 0.014) (Fig. S4a). Collectively, these uncovered that TRPM2-AS physically interacted with miR-612 and may serve as a ceRNA for miR-612.

### TRPM2-AS requires miR-612 to promote GC cell growth and metastasis

Firstly, the expression of miR-612 was greatly downregulated in GC cells (Fig. S4b), and decreased expression of miR-612 was correlated with shorter OS in GC patients (Fig. S4c). Therefore, to determine whether miR-612 plays a tumor suppressive role in GC, we transfected miR-612 mimics or inhibitor into GC cells and validated its suppressive role in GC cell through various experiments (Fig. S4d–i, k–l; Fig. S5a–i).

To find out whether miR-612 mediated the effects of TRPM2-AS on GC cell, we cotransfected miR-612 inhibitor and TRPM2-AS-targeting siRNA into MGC803 cells. Notably, miR-612 partially rescued the effects of TRPM2-AS on the proliferation and metastatic ability of GC cell using CCK8 and transwell assay (Fig. 3k, i; Fig. S5j). Collectively, these data revealed that TRPM2-AS plays an oncogenic role partially through inhibiting the functions of miR-612.

### IGF2BP1 and FOXM1 are downstream targets of miR-612

**Fig. 4:**
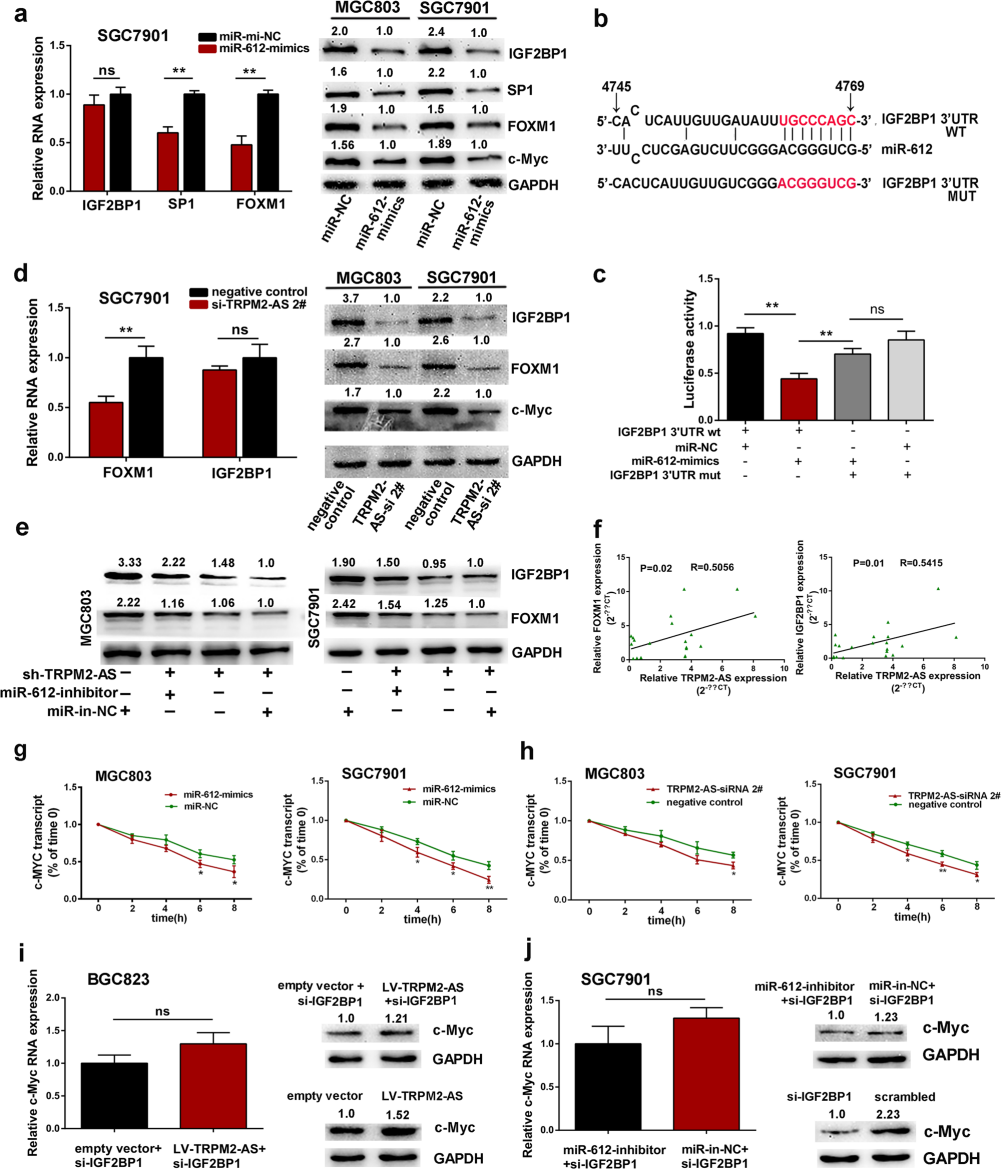
IGF2BP1 and FOXM1 are downstream targets of miR-612. **a** qRT-PCR analysis of the expression of IGF2BP1, SP1, and FOXM1 in SGC7901 cells treated with miR-612 mimics or negative control (left panel). Western blot detection of IGF2BP1, SP1, FOXM1, and c-Myc protein levels in cells transfected with miR-612 mimics or negative control (right panel). NS, not statistically significant. **b** The binding sites of IGF2BP1 3′UTR to miR-612 were mutated. **c** HEK293T cells were cotransfected with miR-612 mimics, negative control and luciferase reporters containing the wild type or mutated transcripts of IGF2BP1 3′UTR as indicated. **d** The RNA level of FOXM1 and IGF2BP1(left panel) and the protein levels of IGF2BP1, FOXM1 and c-Myc (right panel) in GC cells treated with TRPM2-AS-targeting siRNA. **e** Western blot detection of the expression of IGF2BP1 and FOXM1 as indicated. **f**. Pearson correlation analysis between FOXM1 and TRPM2-AS (left), IGF2BP1 and TRPM2-AS (right) in 20 paired GC tissues. **g**, **h** Time course analysis of c-Myc transcript stability in GC cells treated with miR-612 mimics or negative control (**g**) and TRPM2-AS-targeting siRNA (**h**). **i** The expression of c-Myc in BGC823 cells as indicated. **j**. Detection of c-Myc expression in SGC7901 cells transfected with IGF2BP1-targeting siRNA in the presence or absence of miR-612 inhibitor. NS, not statistically significant. Error bars, mean ± SD. *P < 0.05; **P < 0.01.

In order to find the downstream targets of miR-612, online tool like DIANA, miRDB, and RNA22 were used and then combined with analysis of TCGA database. Finally, FOXM1, SP1, IGF2BP1 were identified as candidate targets (Fig. S6b, c). Given the results of previous research, FOXM1 and SP1 are negatively regulated by miR-612^21,22^, and we also confirmed this regulatory role in GC cell (Fig. 4a). FOXM1 was chosen for further research because it showed stronger decline in cell treated with miR-612 mimics. As showed in Fig. 4a, IGF2BP1 was also downregulated by miR-612 mimics, but only in protein level. In order to illustrate the potential interaction between IGF2BP1 and miR-612 further, the 3′UTR wild type sequence of IGF2BP1 harbored the predicted miR-612 binding sites (wt-IGF2BP1) or mutated IGF2BP1 3′UTR sequence (mut-IGF2BP1) were constructed into luciferase reporter vector and then were transfected into HEK293T cells (Fig. 4b). After cotransfected with miR-612 mimics, the wt-IGF2BP1-driven luciferase activity was drastically reduced while it was abolished by the mut-IGF2BP1, which indicated that miR-612 could bind and negatively regulate the expression level of IGF2BP1 (Fig. 4c).

To confirm the ceRNA network between TRPM2-AS, miR-612 and IGF2BP1 or FOXM1, we inhibited the expression of TRPM2-AS and it showed the expression of FOXM1 and IGF2BP1 were both downregulated which was consistent with cells treated by miR-612 mimics (Fig. 4d). Moreover, the suppression of IGF2BP1 and FOXM1 by TRPM2-AS-targeting shRNA was also effectively reversed by miR-612 inhibitor (Fig. 4e). Meanwhile, a positive correlation between TRPM2-AS and IGF2BP1 (*R* = 0.5415, *P* = 0.01) was found in 20 paired gastric cancer tissues, as well as between FOXM1 and TRPM2-AS (*R* = 0.5056, *P* = 0.02) (Fig. 4f). Collectively, these data suggested that TRPM2-AS positively regulates the expression of IGF2BP1 and FOXM1 by impairing miR-612 activity. Besides that, previous evidence disclosed IGF2BP1 could stabilize c-Myc mRNA and thus involved in tumorigenesis^23,24^, so we speculated that TRPM2-AS may also prevent c-Myc mRNA degradation via increasing IGF2BP1 expression. To this end, the expression of TRPM2-AS was downregulated using siRNA, and subsequently c-Myc protein level was drastically decreased (Fig. 4d). Treated with miR-612 mimics also exhibited reduced expression of c-Myc (Fig. 4a). Meanwhile, GC cells were treated with TRPM2-AS-targeting siRNA, miR-612 mimics or negative control and then transcription was repressed using actinomycin D 24 h later. Subsequently, cells were collected for RNA analysis by qPCR according to the time course. The results showed miR-612 enhanced the degradation of c-Myc (Fig. 4g) while TRPM2-AS promoted c-Myc RNA stability (Fig. 4h). On the other hand, in order to determine TRPM2-AS requires IGF2BP1 to regulate c-Myc expression. we inhibited the expression of IGF2BP1 while upregulated TRPM2-AS. As shown in Fig. 4i, the expression of c-Myc was not affected. Meanwhile, in SGC7901 cells treated with IGF2BP1-targeting siRNA in the absence of miR-612 inhibitor or not, the expression of c-Myc was also not changed in these two groups (Fig. 4j).

### IGF2BP1 promotes gastric cancer cell proliferation and metastasis

**Fig. 5:**
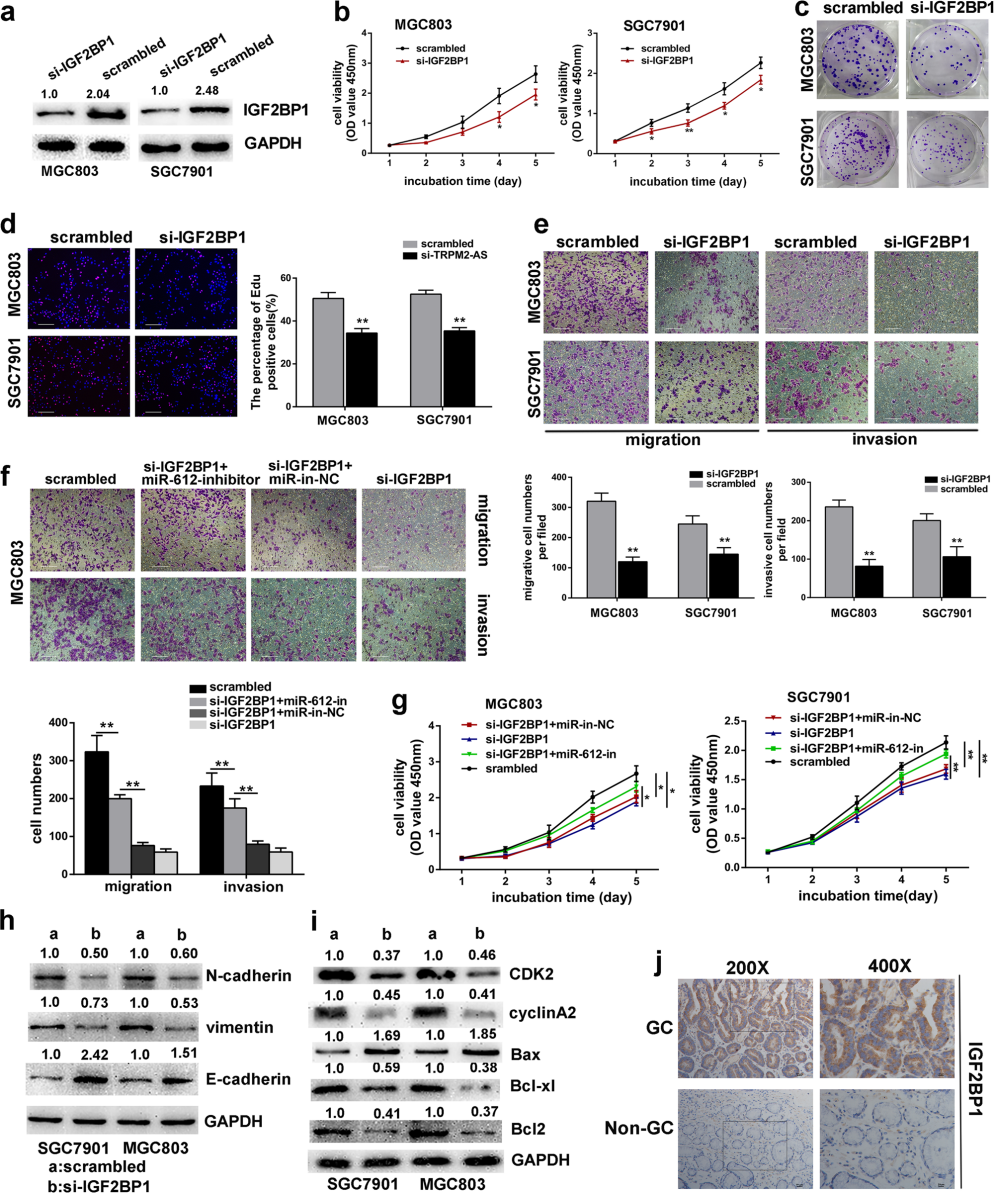
IGF2BP1 promotes GC cell progression. **a** IGF2BP1 was effectively knocked down by siRNA. **b**–**d** The CCK8 assay (**b**), colony formation assay (**c**) and EdU incorporation assay (**d**) were performed to investigate the effects of silencing IGF2BP1 in MGC803 and SGC7901 cells. 100×, scale bar = 100 um. **e** Transwell assays showed knockdown of IGF2BP1 impaired GC cell migration and invasion. 100×, scale bar = 100 um. **f** miR-612 rescue the effects of IGF2BP1 on cell invasion and migration as indicated by transwell assays. scale bar = 100 um. **g** Growth curves for MGC803 cells (left panel) and SGC7901 cells (right panel) as indicated. **h**–**i** the expression of EMT-related proteins (**h**), cell cycle associated proteins and apoptosis-related proteins (**i**) in GC cells transfected with IGF2BP1-targeting siRNA or control. **j** Representative images of the expression and distribution of IGF2BP1 protein in gastric cancer tissue and corresponding normal tissue by IHC. Scale bar = 50 μm (left), scale bar = 25 μm (right). Error bars, mean ± SD. *P < 0.05; **P < 0.01.

Since the effect of IGF2BP1 on gastric cancer has never been reported before, so we transfected IGF2BP1-targeting siRNA into SGC7901 and MGC803 cells to determine whether IGF2BP1 involves in GC cell proliferation and metastasis (Fig. 5a). The CCK8 assay, colony formation and EdU incorporation assay suggested that depletion of IGF2BP1 significantly impaired GC cell proliferation (Fig. 5b–d). In addition, silencing of IGF2BP1 also induced apoptosis accompanied with elevated Bax expression and reduced Bcl2 and Bcl-xl expression, as well as cell cycle arrest at S phase with reduced S-phase checkpoint proteins like CDK2 and cyclinA2 (Fig. 5i; Fig. S7a, b). Otherwise, downregulation of IGF2BP1 also impaired GC cell invasion and migration (Fig. 5e, h). More importantly, immunohistochemistry analysis revealed the high expression and predominant expression in the cytoplasm of IGF2BP1 in GC tissues (Fig. 5j). Overall, these data revealed the oncogenic role of IGF2BP1 in GC. In addition, the CCK8 and transwell assays indicated that inhibition of miR-612 could rescue the suppressive effects on GC cells by IGF2BP1-targeting siRNA (Fig. 5f, g; Fig. S7c). In conclusion, these results suggested there may exist an axis between TRPM2-AS, miR-612 and IGF2BP1 which involved in the tumorigenesis and aggressiveness of gastric cancer.

### TRPM2-AS promotes gastric cancer radioresistance by indirectly regulating FOXM1

**Fig. 6:**
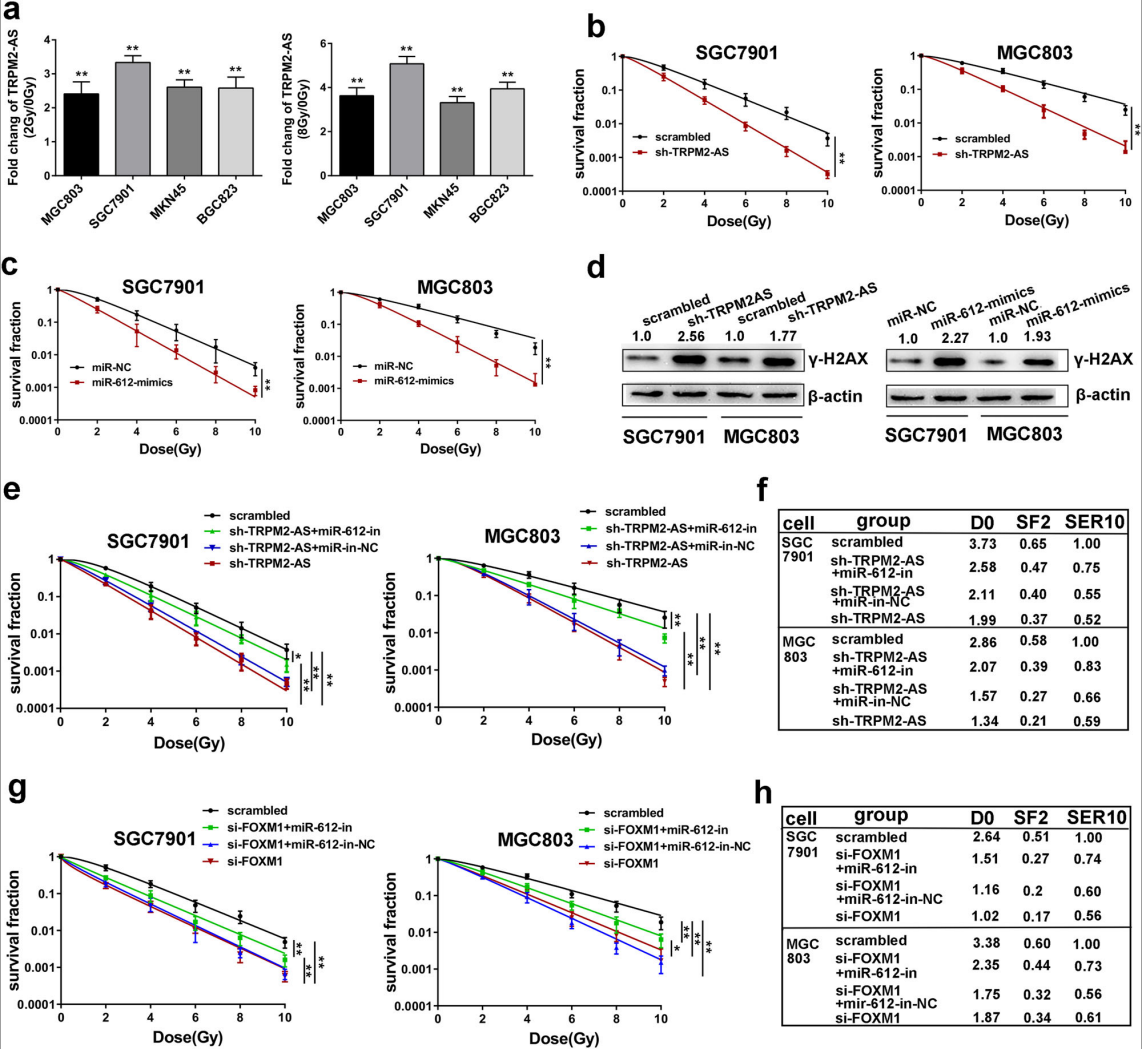
TRPM2-AS promotes GC cell radioresistance by indirectly regulating FOXM1. **a** The expression of TRPM2-AS was upregualted in GC cells received 2 Gy and 8 Gy dose of irradiation compared with paired untreated cells. **b** Radiation survive curves are shown for SGC7901 cells (left) and MGC803 cells (right) which were transduced with TRPM2-AS shRNA or scrambled control with a single increasing dose of 0, 2, 4, 6, 8, 10 Gy. Using ANOVA. **c** Radiation survive curves for SGC7901 cells (left) and MGC803 cells (right) which were transfected with miR-612 mimics or control. Using ANOVA. **d** Western blot analysis of γH2Ax expression in GC cells treated with TRPM2-AS shRNA or scrambled control (right), and treated with miR-612 mimics or control (left) after exposed to 8 Gy dose of radiation. **e** Radiation survive curves for SGC7901 and MGC803 cells cotransfected with TRPM2-AS shRNA, miR-612 inhibitor or negative control as indicated. Using ANOVA. **f** Radiosensitivity parameters of respective cell groups. D0, dose to reduce survival to 37%; SF2, surviving fraction at 2 Gy; SER10, sensitizer enhancement ratio at 10% survival. **g** GC cells were treated as indicated, then Survival fractions were calculated for each group after treatment with increasing dose of radiation. Using ANOVA. **h** Radiosensitivity parameters of respective cell groups. Error bars, mean ± SD. *P < 0.05; **P < 0.01.

In prostate cancer, gene ontology analysis showed TRPM2-AS-depleted cells repressed transcript enrichment in DNA repair^25^, and radioresistance also roles as a significant characteristic of GC, so it is worthy to determining if TRPM2-AS participates in GC radioresistance. First, the expression of TRPM2-AS intensively increased in GC cells exposed to 2 Gy or 8 Gy dose of irradiation compared with untreated paired GC cell lines (Fig. 6a). Then by conducting clonogenic survival assay, the effects of TRPM2-AS on the radiosensitivity of GC cells were investigated. After exposed to increasing dose of irradiation, TRPM2-AS-depleted GC cells showed reduced colony survival fractions (Fig. 6b) and increased expression of DNA damage marker γH2AX after received 8 Gy dose of irradiation (Fig. 6d)^26,27^, which indicating that TRPM2-AS may possess the potential of regulating GC radioresistance. Meanwhile, overexpressing miR-612 showed increased radiosensitivity of GC with reduced colony survival fractions and increased expression of γH2AX (Fig. 6c, d). In view of the interaction between TRPM2-AS and miR-612, rescue experiments further indicated TRPM2-AS may alter the radiation response of GC cell via regulating miR-612 (Fig. 6e). The radiation survival curves include parameters such as D0 (the dose required to reduce survival to 37% of its value), the surviving fraction at 2 Gy (SF2) and the sensitization enhancement ratio at 10% (SER10)^28^. As shown in Fig. 6f, the D0 value was 1.99 and 2.58 for TRPM2-AS shRNA treated group and group cotransfected with miR-612 inhibitor in SGC7901 cell line, respectively. Significantly, FOXM1 was reported to involve in DNA damage–induced senescence program in GC^29^ and whether FOXM1 was a downstream target of miR-612 and TRPM2-AS in regulating GC cell radiosensitivity was investigated. The results showed knockdown of FOXM1 could promote the radiosensitivity of GC cell and this effect was impaired by cotransfected with miR-612 inhibitor (Fig. 6g, h). Hence, above data confirmed that TRPM2-AS may sponge miR-612 and consequently enhanced the expression of FOXM1 to promote GC radioresistance, at least in a part.

**Fig. 7:**
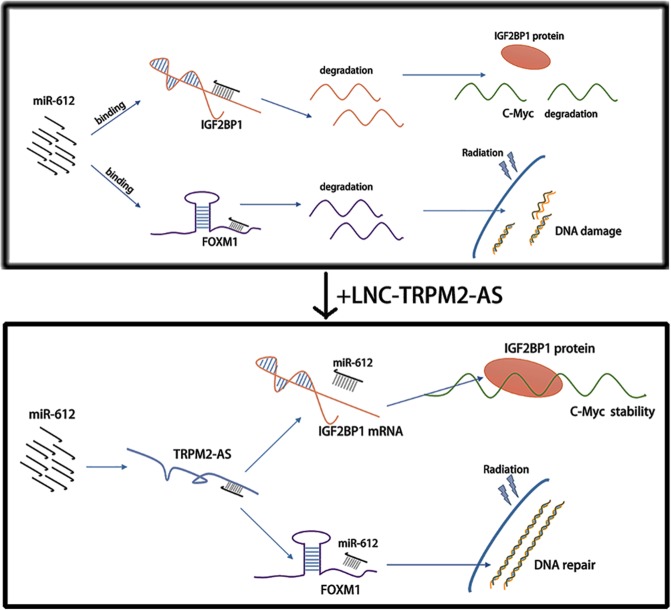
Schematic presentation of possible molecular mechanism underlying the interaction between lncRNA TRPM2-AS and miR-612. TRPM2-AS acted as a ceRNA to sponge miR-612 and upregulated the expression of IGF2BP1 and FOXM1.

In summary, these results indicated that lncRNA TRPM2-AS can directly bind to miR-612 and acting as a sponge to impair miR-612-dependent IGF2BP1 or FOXM1 downregulation in GC (Fig. 7).

## Discussion

Recent studies have revealed the significant role of lncRNA in GC tumorigenesis, suggesting that lncRNAs may be useful diagnostic and therapeutic targets of GC. In this study, we identified a critical GC-associated lncRNA, TRPM2-AS. TRPM2-AS is generated from the antisense of TRPM2^30^, which is an ion channel capable of mediating susceptibility to cell death^31^. Meanwhile, knockdown of TRPM2-AS could upregulate the expression of TRPM2 in GC cell (data not show). However, the relationship between TRPM2 and TRPM2-AS in GC need further researches. Meanwhile, The expression pattern and regulatory mechanism of TRPM2-AS have been reported in melanoma^30^, prostate cancer^25^, lung cancer^32,33^ and breast cancer^34^. Furthermore, TRPM2-AS can promote GC progression by sponging miR-195 and thereby regulate HMGA1 expression^35^. In our study, high expression of TRPM2-AS was correlated with inferior survival and advanced TNM stage. In vivo and in vitro experiments confirmed that TRPM2-AS could promote gastric cancer proliferation and metastasis, which supporting the oncogenic role of TRPM2-AS in GC tumorigenesis.

MicroRNAs are 21–23 nucleotides RNAs which form the RISC complex with Argonaute proteins, and then bind with the 3′UTR of target mRNAs to promote its degradation^36,37^. Recent studies have shown lncRNAs could act as ceRNA to control miRNA availability for its target genes^13,38,39^. This ceRNA model has been certified in different cancer types. For example, lncRNA-PAGBC competitively binds to the tumor suppressive microRNAs miR-133b and miR-511 and therefore titrates miRNAs off their binding sites on SOX4 and PIK3R3, which then activates AKT/mTOR pathway in gallbladder cancer^40^. The results of our study disclosed that TRPM2-AS could serves as a sponge for miR-612 in GC. From previously reported literature, miR-612 was identified as a tumor suppressor. In hepatocellular carcinoma, miR-612 negatively regulates the EMT process through the AKT2 pathway and suppresses the invasive-metastatic cascade^41^. In our study, miR-612 was also confirmed as a tumor suppressor which repressed GC cell proliferation and metastasis. In addition, miR-612 was inversely correlated with TRPM2-AS in human GC tissues. Rescue experiments further showed miR-612 can partially reverse the effects of depletion or overexpression of TRPM2-AS on GC cell. Inhibition of miR-612 also reduced the enrichment of TRPM2-AS in the immunoprecipitates of anti-Ago2. In this regard, such data confirmed that TRPM2-AS could sponge miR-612 to promote GC tumorigenesis.

Finally, FOXM1 and IGF2BP1 were identified as the two downstream targets of miR-612. IGF2BP1 plays essential role in embryogenesis and carcinogenesis, but its function in GC has not been studied yet. In this study, IGF2BP1 was confirmed to play oncogenic role in GC which was negatively regulated by miR-612. IGF2BP1 serves as a RNA binding protein and usually plays its role in a post-transcription level to regulate the expression of certain essential mRNA targets^42,43^. It was reported that c-Myc and IGF2BP1 could form a potential feedback mechanism to reciprocally regulate expression of the other^23^. In this study, we found TRPM2-AS and miR-612 could both regulate the RNA stability of c-Myc and it may be conducted through a TRPM2-AS-miR-612-IGF2BP1-c-MYC axis. Intriguingly, when treated with miR-612 mimics in GC cells, the expression of TRPM2-AS was reduced but its RNA stability was not affected, so we speculated that miR-612 may not regulate the expression of TRPM2-AS independently by promoting its degradation, but through modulating other factors in transcriptional mechanism. Meanwhile, silencing IGF2BP1 could not alter the expression of TRPM2-AS either, which excluded the potential of a positive feedback loop among the three genes (Fig. S6e-h)^44^. FOXM1 is a member of the Forkhead box (FOX) superfamily which serve as transcription factors and was implicated in GC tumorigenesis^45,46^. In recent years, many studies indicated that FOXM1 plays a fundamental role in DNA double-strand breaks repair and negative senescence regulation^47,48^. In GC, it was reported that FOXM1 could negatively regulate cell senescence induced by inhibition of MET signaling or ionizing radiation, and therefore promotes radiation-induced protective response in GC cell^29^. First, the expression of TRPM2-AS was markedly enhanced in GC cells received irradiation. Then the regulatory role and functional relationship of TRPM2-AS and miR-612 to FOXM1 were further confirmed. Collectively, TRPM2-AS was capable to promote GC cell radioresistance and this function was mediated by inhibiting miR-612-dependent downregulation of FOXM1. Except that, the mechanism of how TRPM2-AS-miR-612-FOXM1 axis regulates GC cell radiosensitivity need further exploration and the role of IGF2BP1 in GC radioresistance also demand experimental confirmation.

In conclusion, we identified lncRNA TRPM2-AS as a potential diagnostic and therapeutic target in GC which prompting GC progression and radioresistance. In addition, we revealed that TRPM2-AS served as a microRNA sponge to sequester miR-612 from downstream targets like IGF2BP1 and FOXM1, and therefore increased the expression of c-Myc by enhancing IGF2BP1 and promoted GC cell radioresistance by inducing the upregulation of FOXM1. Thus, the pleiotropic roles of TRPM2-AS on GC tumorigenesis and therapy resistance suggest that it could be a useful target for clinically application in GC.

## Materials and methods

### Tissue samples

Gastric cancer tissues and corresponding adjacent non-tumor tissues were obtained from 80 patients with GC who underwent radical gastrectomy at the Department of General Surgery, The First Affiliated Hospital of Nanjing Medical University (NMU), China. All collected tissue samples were immediately snap frozen in liquid nitrogen and stored at −80 °C until required. This study was approved by the Ethics Committee of the First Affiliated Hospital of NMU. Written informed consent was signed before specimen collection.

### Cell lines

Five human GC cell lines, SGC7901, BGC823, MKN45, MGC803, HGC27 and the normal human gastric mucous epithelium cell line GES-1 which studied in this research were obtained from the Cell Bank of the Chinese Academy of Science (Shanghai, China). Above GC Cells and GES-1 were cultured in RPMI-1640 medium supplemented with 10% fetal bovine serum (Wisent, Montreal, Canada) and 1% antibiotics (100 U/ml penicillin G and 100 mg/ml streptomycin), HEK-293T cells were cultured in Dulbecco’s modified Eagle’s medium supplemented with 10% FBS and 1% penicillin-streptomycin. Cells were incubated in a humidified atmosphere at 37 °C containing 5% CO2. All cells were authenticated by short tandem repeat analysis and passed for less than 6 months in culture when the experiments were performed.

### RNA extraction and quantitative RT-PCR assays

Total RNA was extracted from tissues or cultured cells using TRIzol reagent (Invitrogen, Carlsbad, CA, USA) according to the manufacturer’s instructions. Then qRT-PCR assays were performed according to previous procedures^49^. Results were standardized to the expression of β-actin or GAPDH. MiRNAs were reverse transcribed after polyadenylation using Revert Aid First Strand cDNA Synthesis Kit (Thermo Scientific, MA, USA). Levels of miRNA expression were standardized to U6. Specific primers used in this study were summed at Supplementary Table 2. All procedures were carried out in triplicate and relative expression was calculated by the 2^−ΔΔCT^ method.

### Cell proliferation, colony formation, and 5-Ethynyl-2′-deoxyuridine (EdU) incorporation assay

Cell counting kit 8(CCK8) assay, colony formation assay and EdU incorporation assay were used to determine the effects of corresponding genes to the proliferation of gastric cancer cell, the process were described before^49^.

### Wound scratch and transwell assays

To determine the alternation of gastric cancer cell migration and invasion, we performed wound scratch and transwell assays following previous protocol^49^.

### Cell transfection and vector construction

In this study, siRNAs, miRNA mimics or inhibitor and corresponding controls were used (Genepharma, Shanghai, China). The nucleotide sequences of these oligonucleotides are shown in Supplementary Table 3. Oligonucleotides (50 nm) were transfected into GC cells using Lipofectamine 3000 (Invitrogen, CA, USA) in accordance with the manufacturer’s protocol. At 48 h post transfection, cells were harvested for qRT-PCR or Western blot analysis. TRPM2-AS 875 bp cDNA sequences were subcloned and amplified into the lentiviral expression vector (Genepharma, Shanghai, China). Biologically active short hairpin RNAs (shRNA) targeting TRPM2-AS was generated using another lentiviral expression vector (Genechem, Shanghai, China). GC cells were then infected with lentiviral expression vector at a suitable multiplicity of infection (MOI) when they grew to 30–40% confluence. Stable cell lines were obtained by using 2 μg/ml puromycin (Sigma-Aldrich, St-Louis, Missouri, USA) for about three weeks.

### Flow cytometric analysis

Flow cytometric analysis was conducted to investigate the cell cycle distribution and the percentage of apoptotic cells. The detailed procedures were described in previous^50^. The experiments were performed in triplicate.

### Western blot assay and antibodies

Western blot assay was applied in accordance with the standard conditions^49^. The primary antibody used were listed in Supplementary Table 4.

### Animal experiments

Fifteen four weeks old female BALB/c nude mice were purchased from Animal Center of NMU and all experimental animals were in accordance with NMU Institutional Animal Care and Use Committee. For the tumorigenicity studies, SGC7901 and MGC803 cells which were stably transfected with control vector or TRPM2-AS shRNA (1 × 10^6^ cells/100 μl PBS) were subcutaneously injected into the flank of the randomly assigned nude mice. The tumor volume was measured every one week and calculated by the formula: volume = (length × width^2^)/2. At 28 days after injection, mice were euthanized. BGC823 cells transfected with LV-TRPM2-AS or control empty vector followed the same step as mentioned above. For the metastasis model, fourteen female nude mice were randomly divided into two groups which were injected with control vector or TRPM2-AS shRNA (1 × 10^6^ cells/100 μl PBS) into the tail vein. 4 weeks later, mice were euthanized and the lung were subjected to hematoxylin and eosin staining while clone numbers were counted.

### FISH and subcellular fractionation assay

A Cy3-labeled TRPM2-AS complementary DNA probe mix (RiBo Ltd, Guangzhou, China) was synthesized in vitro. In accordance with the protocol, GC cells were plated into a 15 mm confocal dish at a density of 20,000 cells/dish with RPMI 1640 (10% FBS) for 24 h. Next day, the cells were fixed and permeabilized. After blocked with 200 μl pre-hybridization buffer, 20 μm probe mix was blended with the hybridization buffer and then added into the dishes to hybridize with the target sequence overnight. At the second day, the dishes were treated with washing buffer to reduce the background signal. Finally, the nuclei were stained by DAPI and photographed by confocal microscope. Cytoplasmic and nuclear RNA were separated and purified using the Cytoplasmic and Nuclear RNA Purification kit (Norgen, Thorold, ON, Canada) according to the manufacturers’ instructions.

### Dual luciferase reporter assay

To determine the binding among lncRNA, miRNA and mRNA, the complementary DNA sequence were subcloned into pGL3-basic luciferase reporter vector (Promega, Madison, Wisconsin, USA). HEK-293T cells (1 × 10^5^) were seeded in 24-well plates for 24 h before transfection, and then together with miRNA mimics or control, the luciferase reporter vectors were transfected into HEK 293 T cells. After 48 h of incubation, the luciferase activities were measured using a Dual-Luciferase Reporter Assay System (Promega, Madison, Wisconsin, USA) according to manufacturer’s instructions. Relative luciferase activity was normalized to *Renilla* luciferase.

### RNA immunoprecipitation

For exploring whether TRPM2-AS participates in miRNA mediated RISC complex, RIP assays were applied with anti-Ago2 antibody (Abcam, CA, MA, USA) using the Magna RIPTM RNA-binding protein immunoprecipitation kit (Millipore, Bedford, MA, USA) according to the manufacturer’s instruction. Briefly, GC cells were lysed and incubated with anti-AGO2 antibodies at 4 °C overnight, then added protein A magnetic beads and incubated for another 4 h. The coprecipitated RNAs were then extracted and detected by qRT-PCR.

### RNA half-life analysis

Briefly, SGC7901 and MGC803 cells were seeded into 6-well plates at a density of 5 × 10^5^ cells/well and cultured for 24 h to reach 70% confluency. To inhibit transcriptional activity, 5 mg/ml of actinomycin D (Sigma-Aldrich, St-Louis, Missouri, USA) were added to the medium. Cells were harvested at 0 h, 2 h, 4 h, 6 h, 8 h, then analyzed by qRT-PCR.

### Immunohistochemistry

Immunohistochemistry (IHC) were performed as previously described^49^.

### Clonogenic survival assay

GC cells were seeded into 6-well plates at a density of 200, 500, 1000, 2000, 4000, 8000 cells/well. Twenty-four hours later, the cells were treated with a single dose of 0, 2, 4, 6, 8, 10 Gy X-ray irradiation from a medical linear accelerator (Precise accelerator, Elekta, Sweden) at room temperature, respectively. Then cultured for 14 days later, the cells were stained with 1% crystal violet and the numbers of colonies >50 cells were counted. Individual assays were performed with triplicate wells, and repeated at least three times. Surviving fraction (SF) was estimated by the following formula: SF = [number of colonies formed/number of cells seeded × plating efficiency of the control group], where plating efficiency was calculated as the ratio between colonies observed and number of cells plated of control group. A survival curve was derived using the following model: SF = 1 − (1 − e^(−K×D)^)^*N*^.

### Bioinformatic analyses

The downstream miRNA targets of Lnc-TRPM2-AS were predicted using RNA22-HAS (https://cm.jefferson.edu/rna22/) and RegRNA2.0 (http://regrna2.mbc.nctu.edu.tw/). DIANA (http://diana.imis.athena-innovation.gr/DianaTools), miRDB (http://mirdb.org/cgi-bin/) and RNA22-HAS were used to find the targets of miR-612.

### Statistics analysis

All statistical analyses were performed using SPSS v19.0 and GraphPad Prism 6. For statistical comparisons, one-way analysis of variance, wilcoxon test and two-tailed Student’s *t*-tests were performed as appropriate. The data are expressed as the mean ± standard deviation (SD) unless otherwise specified, all of the experiments in our study were independently performed in triplicate, *p* < 0.05 was considered statistically significant.

## Supplementary information

Legends of Suppl Information

Suppl Fig.S1

Suppl Fig.S2

Suppl Fig.S3

Suppl Fig. S4

Suppl Fig.S5

Suppl Fig. S6

Suppl Fig.S7

Suppl Table 1

Suppl Table 2

Suppl Table 3

Suppl Table 4
