# Supplementary material for: Long noncoding RNA TRPM2-AS acts as a microRNA sponge of miR-612 to promote gastric cancer progression and radioresistance
Source: Oncogenesis. 2020 Mar 2;9(3):29. doi: 10.1038/s41389-020-0215-2 (PMC7052141; doi:10.1038/s41389-020-0215-2)
Supplement: Supplementary file 1 — Legends of Suppl Information [file 41389_2020_215_MOESM1_ESM.docx]

Long noncoding RNA TRPM2-AS acts as a microRNA sponge of miR-612 to promote gastric cancer progression and radioresistance

Jian Xiao^1, *^, Linling Lin^1, *^, Dakui Luo^3, *^, Liang Shi^1, *^, Wangwang Chen^1, *^, Hao Fan^1^, Zengliang Li^1^, Xiang Ma^1^, Peidong Ni^1^, Li Yang^1,2^, Zekuan Xu^1^

^1^ Department of General Surgery, The First Affiliated Hospital of Nanjing Medical University, Nanjing, Jiangsu Province, China;

^2^ Department of General Surgery, Liyang People’s Hospital, Liyang Branch Hospital of Jiangsu Province Hospital, Liyang, Jiangsu Province, China;

^3^ Department of Colorectal Surgery, Fudan University Shanghai Cancer Center, 270 Dong An Road, Shanghai 200032, China

Legends of supplementary information

**Fig. S1.** The coding potential of TRPM2-AS is very low. **a.** Coding potential of TRPM2-AS from CAPT. **b.** Coding potential of TRPM2-AS in CPC. **c.** TRPM2-AS failed to generate a protein more than 165 amino acid.

**Fig. S2.** Ablation of TRPM2-AS induces apoptosis in GC cells. **a.** The expression of TRPM2-AS was downregulated by shRNA targeting TRPM2-AS. **b.** The apoptotic rate in GC cells transfected with TRPM2-AS-targeting siRNA or negative control. **c.** the formation of apoptotic blebs (pointed by arrows). scale bar=50um. **d.** The expression level of EMT-related and cell cycle-related protein (left panel), apoptosis-related proteins (right panel) after silencing TRPM2-AS. Grayscale value was presented. Error bar, mean±SD; *, p<0.05; **, p<0.01.

**Fig. S3.** Ectopic expression of TRPM2-AS in BGC823 cells promotes cell proliferation, migration and invasion. **a.** Exogenous expression of TRPM2-AS by lentivirus vector. **b-d.** CCK8 **(b)**, colony formation **(c)** and EdU assays **(d)** were performed to determine the proliferation of BGC823 cells.100×, scale bars=100um. **e and f.** Wound scratch and transwell assays were used to examine the effects of ectopic expression of TRPM2-AS on cell migration and invasion. 100×, scale bars=100um. **g-i.** The tumor volume and the tumor weight of transplanted tumors were monitored. **j.** Cell cycle distribution in BGC823 cells. **k.** The metastatic animal model was established by injecting SGC7901 cells which transduced with TRPM2-AS-targeting shRNA or control into the tail vein of nude mice. lungs with tumor nodule from mice in each group are shown. Error bars, mean±SD.*, P < 0.05; **, P < 0.01.

**Fig. S4.** miR-612 repressed the proliferation and metastasis of SGC7901 cell. **a.** the expression of miR-612 negatively correlates with TRPM2-AS in 20 paired GC tissues. Using pearson correlation analysis. **b.** The expression of miR-612 in GC cell line compared with GES-1. **c.** Kaplan-Meier analysis showed low expression of miR-612 was correlated with poor overall survival rate using online database Kaplan Meier-plotter. **d-f.** miR-612 impaired the proliferation of SGC7901 cells by conducting CCK8 assay(**d**), Colony formation assays(**e**) and EdU incorporation assays(**f**). Scale bar=100um. **g and h.** Transwell assay suggested that ectopic expression of miR-612 damaged the migration and invasion of SGC7901 cells. scale bar=100um. **i.** SGC7901 cells transfected with miR-612 mmics or negative control were subjected to wound scratch assays. **j.** Analysis of BGC823 cell migration and invasion as indicated. scale bar =100um. **k and l.** Flow cytometric analysis and western blot detection of cell cycle distribution (**k**) and apoptotic rate (**l**). Error bar, mean±SD; *, p<0.05; **, p<0.01.

**Fig. S5.** miR-612 serves as a tumor suppressor in GC. **a-b.** GC cells were transfected with miR-612 mimics**(a)** or miR-612 inhibitor**(b)**. **c-d.** CCK8**(c)**, colony formation **(d)** and EdU incorporation assays **(e)** were performed to examine GC cell proliferation. 100×, Scale bars=100um. **f and g.** Wound scratch assays were performed to investigate the effects of miR-612 on GC cell migration. **h.** Effects of miR-612 on GC cell migration and invasion by conducting tanswell assays. 100×, Scale bars=100um. **i.** Western blot analysis of N-cadherin, E-cadherin, vimentin protein level. **j.** MGC803 cells cotransfected with TRPM2-AS siRNA, miR-612 inhibitor or negative control were subjected to transwell assay as indicated. 100×, Scale bars=100um. Error bars, mean±SD.*, P < 0.05; **, P < 0.01.

**Fig. S6.** IGF2BP1 and FOXM1 are downstream targets of miR-612. **a.** Kaplan-Meier OS curves according to the expression levels of IGF2BP1 by using online database Kaplan Meier-plotter. log rank test, p=0.021. **b.** Relative expression of IGF2BP1 in TCGA database. **c.** The correlation between TRPM2-AS and IGF2BP1 in GC patients from TCGA cohort (left) and the correlation between TRPM2-AS and FOXM1 in TCGA cohort of GC (right). Using pearson correlation analysis. **d.** Western blot analysis of FOXM1 and IGF2BP1 in 10 xenograft tumor tissue samples. **e.** miR-612 mimics downregualted the expression of TRPM2-AS in GC cells. **f.** Analysis of the RNA stability of TRPM2-AS. **g.** The expression of TRPM2-AS in GC cells treated with IGF2BP1-targeting siRNA. **h.** Time course analysis of TRPM2-AS transcript stability in SGC7901 cells treated with IGF2BP1 siRNA or negative control. Error bar, mean±SD; *, p<0.05; **, p<0.01.

**Fig. S7.** IGF2BP1 induced cell cycle arrest and apoptosis in GC cells. **a.** Flow cytometric analysis of apoptotic rate in GC cells transfected with IGF2BP1-targeting siRNA or negative control. **b.** cell cycle analysis in GC cells treated with IGF2BP1-targeting siRNA or negative control. c. Transwell assays were performed in SGC7901 cells as indicated. 100×.Error bar,mean±SD; *, p<0.05;**, p<0.01.
