# Supplementary figures and images for: Long noncoding RNA TRPM2-AS acts as a microRNA sponge of miR-612 to promote gastric cancer progression and radioresistance
Source: Oncogenesis. 2020 Mar 2;9(3):29. doi: 10.1038/s41389-020-0215-2 (PMC7052141; doi:10.1038/s41389-020-0215-2)

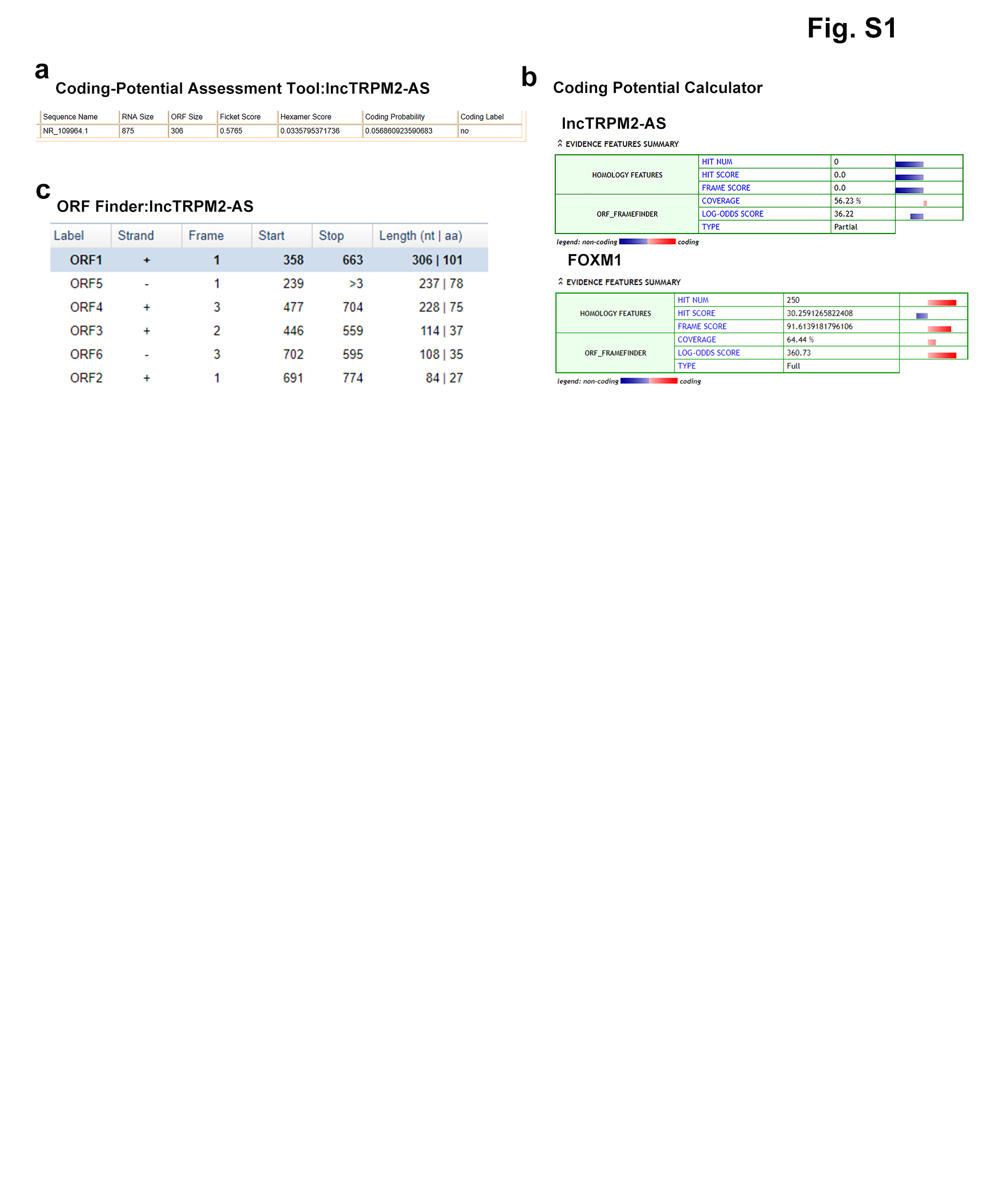

Supplement: Supplementary file 2 — Suppl Fig.S1 [file 41389_2020_215_MOESM2_ESM.tif]

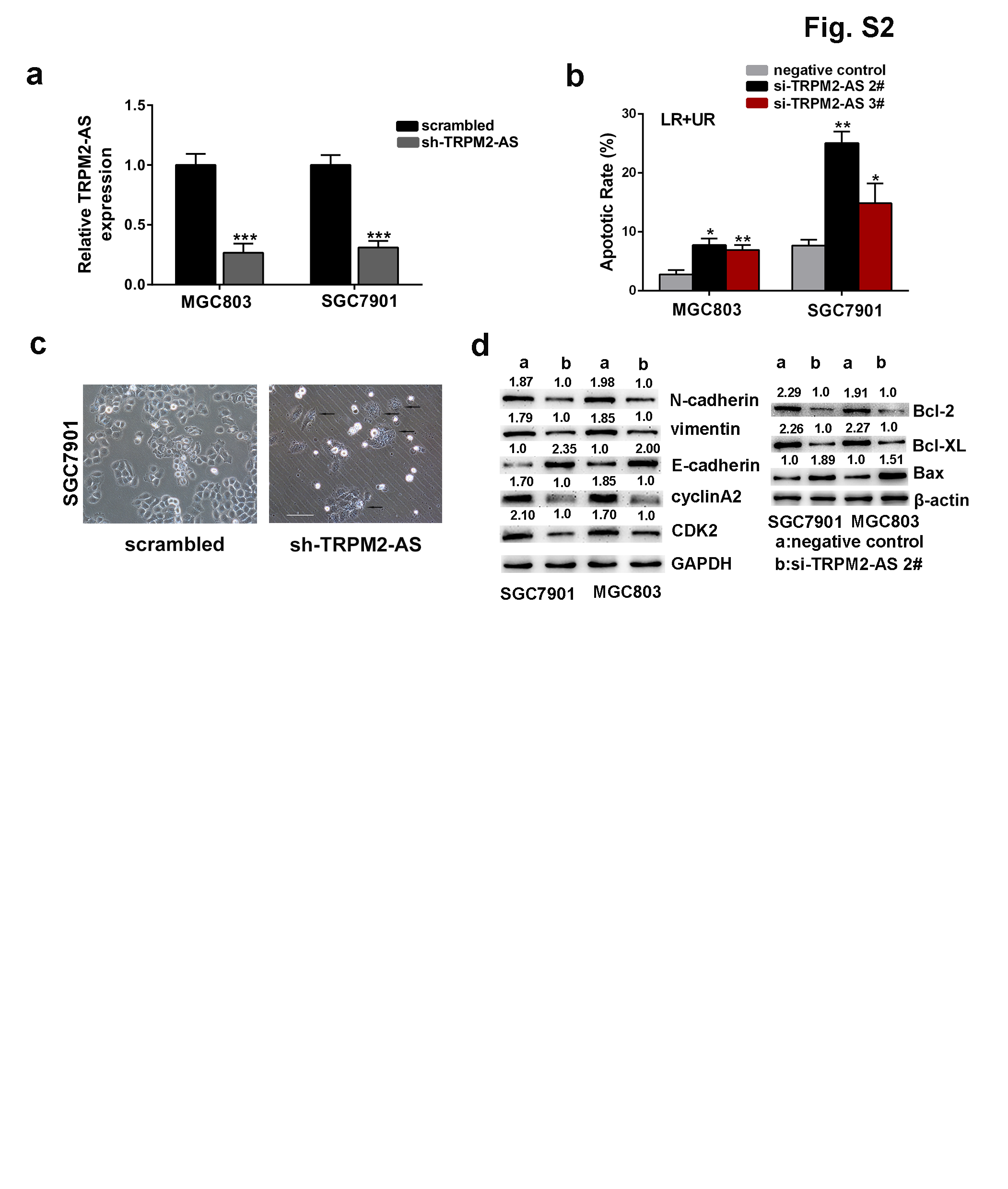

Supplement: Supplementary file 3 — Suppl Fig.S2 [file 41389_2020_215_MOESM3_ESM.tif]

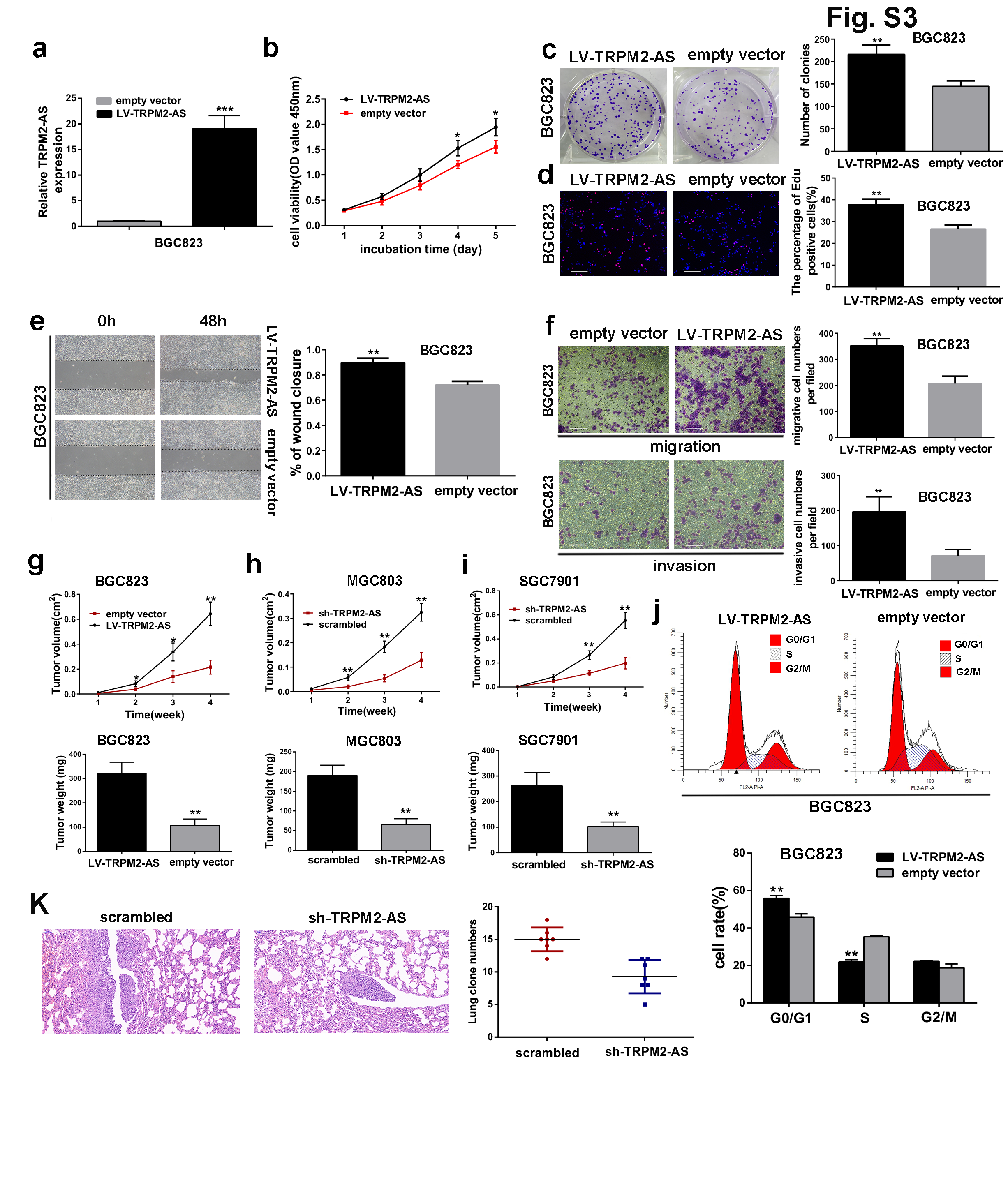

Supplement: Supplementary file 4 — Suppl Fig.S3 [file 41389_2020_215_MOESM4_ESM.tif]

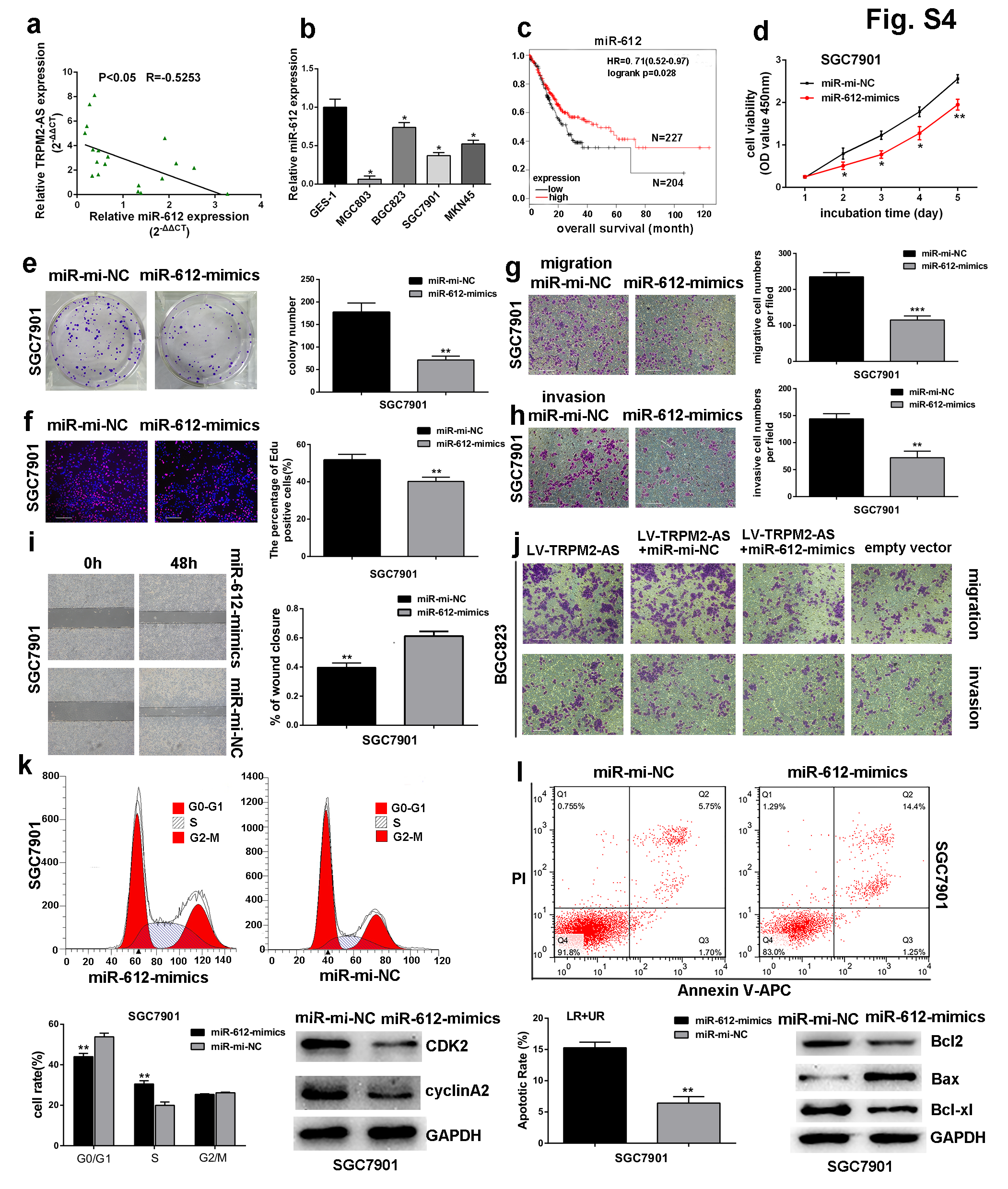

Supplement: Supplementary file 5 — Suppl Fig. S4 [file 41389_2020_215_MOESM5_ESM.tif]

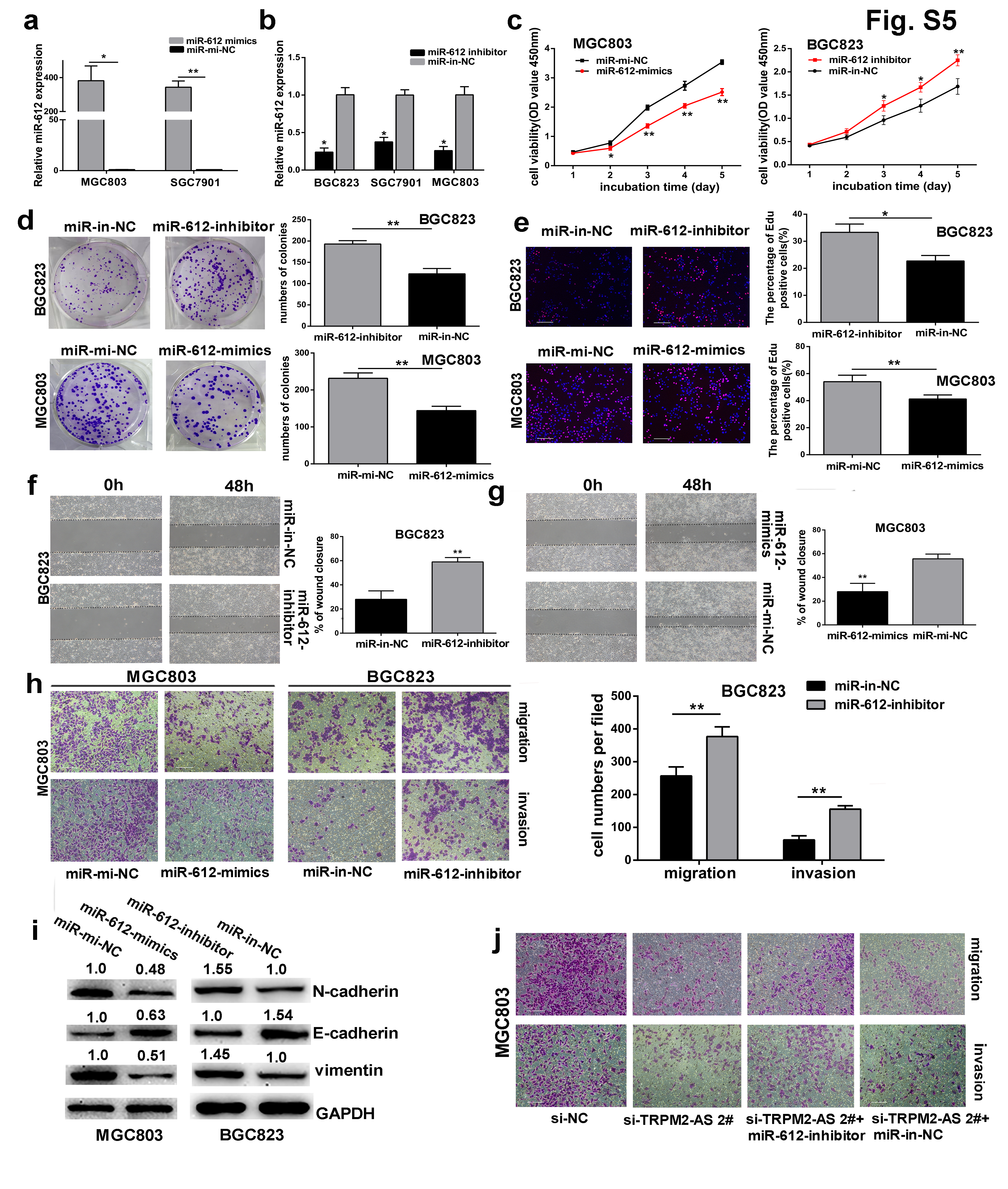

Supplement: Supplementary file 6 — Suppl Fig.S5 [file 41389_2020_215_MOESM6_ESM.tif]

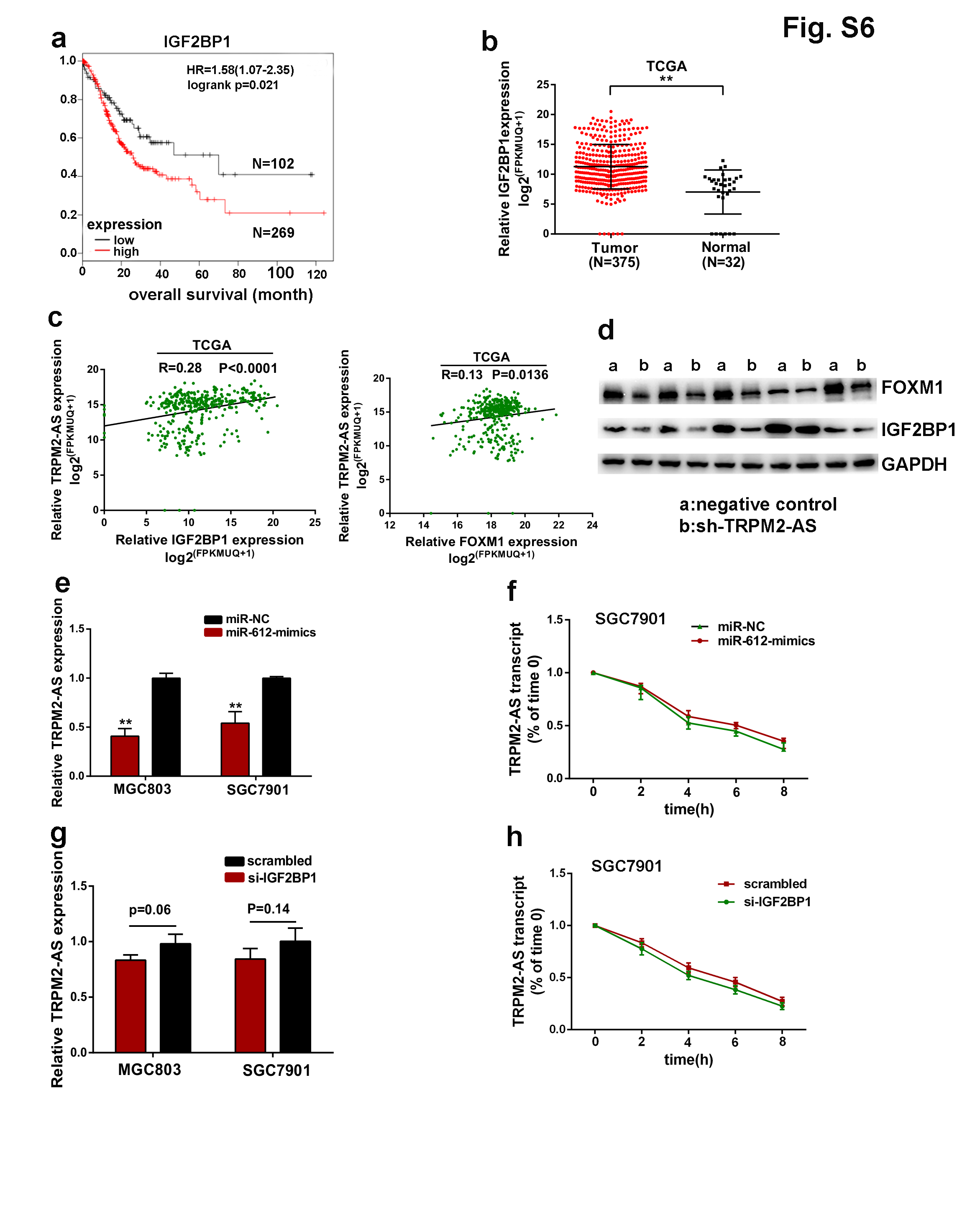

Supplement: Supplementary file 7 — Suppl Fig. S6 [file 41389_2020_215_MOESM7_ESM.tif]

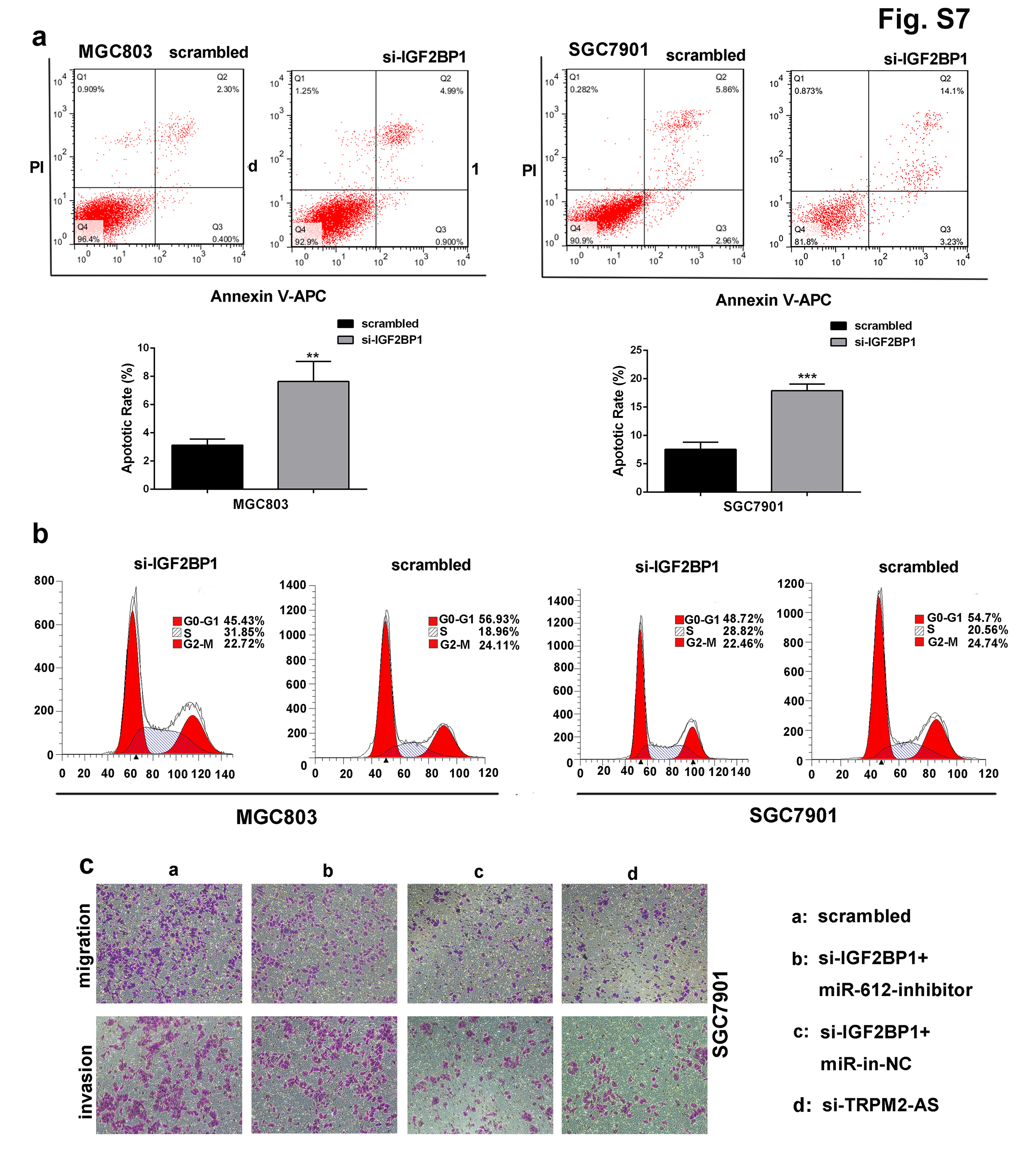

Supplement: Supplementary file 8 — Suppl Fig.S7 [file 41389_2020_215_MOESM8_ESM.tif]
