# Supplementary material for: Long noncoding RNA TRPM2-AS acts as a microRNA sponge of miR-612 to promote gastric cancer progression and radioresistance
Source: Oncogenesis. 2020 Mar 2;9(3):29. doi: 10.1038/s41389-020-0215-2 (PMC7052141; doi:10.1038/s41389-020-0215-2)
Supplement: Supplementary file 9 — Suppl Table 1 [file 41389_2020_215_MOESM9_ESM.docx]

**Supplementary Table 1. The correlation between the expression level of TRPM2-AS and clinicopathological features in GC patients.**

| Characteristics | Group | Cases | Expression of TRPM2-AS* | p-value |
| --- | --- | --- | --- | --- |
| Age | <60 | 34 | 5.915±1.1171 | p=0.509 |
|  | ≥60 | 46 | 5.011±0.8288 |  |
| Gender | Male | 60 | 4.737±0.6276 | p=0.196 |
|  | Female | 20 | 7.370±1.8877 |  |
| T stage | Tis-T1 | 5 | 0.294± 0.4934 | p<0.001 |
|  | T2-T4 | 75 | 5.735±0.6974 |  |
| Lymph node metastasis | Absent(N0) | 14 | 2.093±0.76583 | P=0.001 |
|  | Present(N1-N3) | 66 | 6.095±0.7704 |  |
| TNM stage | I-II | 8 | 1.703±0.7325 | P=0.001 |
|  | III-IV | 72 | 5.805±0.7250 |  |
| Location | Cardia | 51 | 5.826± 0.7994 | P=0.405 |
|  | Non-cardia | 29 | 4.637±1.2506 |  |
| Total |  | 80 |  |  |

**Students T test; *, mean±SE, 2^-ΔΔCT^.**
