# Supplementary material for: Long noncoding RNA TRPM2-AS acts as a microRNA sponge of miR-612 to promote gastric cancer progression and radioresistance
Source: Oncogenesis. 2020 Mar 2;9(3):29. doi: 10.1038/s41389-020-0215-2 (PMC7052141; doi:10.1038/s41389-020-0215-2)
Supplement: Supplementary file 10 — Suppl Table 2 [file 41389_2020_215_MOESM10_ESM.doc]

**Supplementary Table 2. Primers used for Quantitative RT-PCR**

| RNA | 5'to 3' |
| --- | --- |
| TRPM2 | Forward CCATCCGTGACCTTCTCATT |
|  | Reverse CTCTGAGCCCAGATGATTCC |
| TRPM2-AS | Forward CCAGGAACCAGAACCAAACT |
|  | Reverse TGTCCGTCTGCTGAGACATC |
| IGF2BP1 | Forward CAGGAGATGGTGCAGGTGTTTATCC |
|  | Reverse GTTTGCCATAGATTCTTCCCTGAGC |
| FOXM1 | Forward CGTGGATTGAGGACCACTTT |
|  | Reverse GGCTTAAACACCTGGTCCAA |
| SP1 | Forward GTGGGAAGCCAAGACAACAT |
|  | Reverse GGGAGGAGGAAGACCATTCT |
| c-Myc | Forward GGGCTTTATCTAACTCGCTGTA |
|  | Reverse GCTATGGGCAAAGTTTCGTG |
| β-actin | Forward GCATCGTCACCAACTGGGAC |
|  | Reverse ACCTGG CCGTCAGGCAGCTC |
| GAPDH | Forward TGCACCACCAACTGCTTAGC |
|  | Reverse GGCATGGACTGTGGTCATGAG |
| Universal reverse primer | GCGAGCACAGAATTAATACGAC |
| U6 | Forward CTCGCTTCGGCAGCACA |
|  | Universal reverse primer |
| miR-612 | Forward GCAGGGCTTCTGAGCTCCTTAA |
|  | Universal reverse primer |
| miR-103a-2-5p | Forward AGCTTCTTTACAGTGCTGCCTTG |
|  | Universal reverse primer |
| miR-125b-5p | Forward ACACTCCAGCTGGGTCCCTGAGACCCTAAC |
|  | Universal reverse primer |
| miR-138-5P | Forward AGCTGGTGTTGTGAATCAGGCCG |
|  | Universal reverse primer |
| miR-34a-5p | Forward TGGCAGTGTCTTAGCTGGTTGT |
|  | Universal reverse primer |
