# Supplementary material for: Long noncoding RNA TRPM2-AS acts as a microRNA sponge of miR-612 to promote gastric cancer progression and radioresistance
Source: Oncogenesis. 2020 Mar 2;9(3):29. doi: 10.1038/s41389-020-0215-2 (PMC7052141; doi:10.1038/s41389-020-0215-2)
Supplement: Supplementary file 11 — Suppl Table 3 [file 41389_2020_215_MOESM11_ESM.doc]

**Supplementary Table3. siRNA and shRNA used in this study**

| siRNA | 5'to 3' |
| --- | --- |
| si-TRPM2-AS-1 | sense GGGAAGAUGUCUCAGCAGACG |
|  | antisense UCUGCUGAGACAUCUUCCCCU |
| si-TRPM2-AS-2 | sense AGACCUAUGAGGAGACAUAAC |
|  | antisense UAUGUCUCCUCAUAGGUCUCG |
| si-TRPM2-AS-3 | sense CGAACCUUCCCUAAUAGAAAC |
|  | antisense UUCUAUUAGGGAAGGUUCGGG |
| siRNA-negative control | sense UUCUCCGAACGU GUC ACGUTT |
|  | antisense ACGUGACACGUUCGGAGAATT |
| miR-612 inhibitor | AAGGAGCUCAGAAGCCCUGCCCAGC |
| microRNA inhibitor NC | CAGUACUUUUGUGUAGUACAA |
| miR-612 mimics | sense GCUGGGCAGGGCUUCUGAGCUCCUU |
|  | antisene GGAGCUCAGAAGCCCUGCCCAGCUU |
| microRNA mimics NC | sense UUCUCCGAACGU GUC ACGUTT |
|  | antisense ACGUGACACGUUCGGAGAATT |
| si-IGF2BP1 | sense GCUCCCUAUAGCUCCUUUATT |
|  | antisense UAAAGGAGCUAUAGGGAGCTT |
| si-FOXM1 | sense UGGUUAAUAAUCUUGAUCCCA |
|  | antisense GGAUCAAGAUUAUUAACCACC |
| shRNA | 5'to 3' |
| TRPM2-AS shRNA | AGACCTATGAGGAGACATAAC |
| TRPM2-AS scrambled NC | TTCTCCGAACGTGTCACGT |
