# Supplementary material for: Long noncoding RNA TRPM2-AS acts as a microRNA sponge of miR-612 to promote gastric cancer progression and radioresistance
Source: Oncogenesis. 2020 Mar 2;9(3):29. doi: 10.1038/s41389-020-0215-2 (PMC7052141; doi:10.1038/s41389-020-0215-2)
Supplement: Supplementary file 12 — Suppl Table 4 [file 41389_2020_215_MOESM12_ESM.doc]

**Supplementary Table 4. Antibodies used for Western blot**

| antibody | company/provider(Dilution ratio) |
| --- | --- |
| GAPDH | CST, Danvers, MA, USA(1:5000) |
| β-actin | CST, Danvers, MA, USA(1:5000) |
| anti-N-Cadherin | Proteintech,Wuhan,China(1:2000) |
| anti-IGF2BP1 | Proteintech,Wuhan,China(1:2000) |
| anti-FOXM1 | Proteintech,Wuhan,China(1:2000) |
| anti-SP1 | Proteintech,Wuhan,China(1:2000) |
| anti-CyclinA | Abcam,CA,MA,USA(1:2000) |
| anti-CDK2 | Abcam,CA,MA,USA(1:2000) |
| anti E-Cadherin | Abcam,CA,MA,USA(1:2000) |
| anti-Vimentin | Abcam,CA,MA,USA(1:2000) |
| anti-γH2AX | ABclonal,Wuhan,China(1:2000) |
| anti-Bcl-2 | CST, Danvers, MA, USA(1:2000) |
| anti-Bcl-xl | CST, Danvers, MA, USA(1:2000) |
| anti-Bax | CST, Danvers, MA, USA(1:2000) |
| anti -Ago2 | Abcam,CA,MA,USA(1:2000) |
| anti-c-Myc | CST, Danvers, MA, USA(1:2000) |
